# Reconciliation of Gene and Species Trees

**DOI:** 10.1155/2014/642089

**Published:** 2014-03-27

**Authors:** L. Y. Rusin, E. V. Lyubetskaya, K. Y. Gorbunov, V. A. Lyubetsky

**Affiliations:** ^1^Institute for Information Transmission Problems (Kharkevich Institute), Russian Academy of Sciences, Bolshoy Karetny Pereulok 19, Moscow 127994, Russia; ^2^Faculty of Biology, Moscow State University, Leninskie Gory 1-12, Moscow 119234, Russia

## Abstract

The first part of the paper briefly overviews the problem of gene and species trees reconciliation with the focus on defining and algorithmic construction of the evolutionary scenario. Basic ideas are discussed for the aspects of mapping definitions, costs of the mapping and evolutionary scenario, imposing time scales on a scenario, incorporating horizontal gene transfers, binarization and reconciliation of polytomous trees, and construction of species trees and scenarios. The review does not intend to cover the vast diversity of literature published on these subjects. Instead, the authors strived to overview the problem of the evolutionary scenario as a central concept in many areas of evolutionary research. The second part provides detailed mathematical proofs for the solutions of two problems: (i) inferring a gene evolution along a species tree accounting for various types of evolutionary events and (ii) trees reconciliation into a single species tree when only gene duplications and losses are allowed. All proposed algorithms have a cubic time complexity and are mathematically proved to find exact solutions. Solving algorithms for problem (ii) can be naturally extended to incorporate horizontal transfers, other evolutionary events, and time scales on the species tree.

## 1. Reconciliation of Gene and Species Trees: A Brief Overview 

This section of the paper does not intend to cover the vast diversity of published literature on the problem of trees reconciliation. Instead, the authors strived to overview the problem of defining algorithmic construction of the evolutionary scenario as a central concept in many areas of evolutionary research. Important definitions are discussed, and essential problems are highlighted. We believe that, despite many approaches to defining the scenario known today, its solid theoretical framework is still to be developed.

### 1.1. Evolutionary Scenarios and Fields of Their Application

The evolution of the genome, apart from the mutation process, is an entangled complex of individual and concerted evolutions of genes, their regulations, gene content and arrangement on chromosomes, genetic flows between the genome and intracellular organelles, and so forth. Their evolutionary histories often do not coincide with each other and with patterns of speciation giving the rise to a variety of evolutionary events, such as gene duplications, losses, gains, horizontal transfers, chromosome rearrangements, and others. These phenomena play a pivotal role in evolutionary plasticity of the genome, the emergence of genes and gene families with novel functions, maintenance of the molecular machinery of the cell, evolutionary adaptation of the organism, and so forth. As known today, various types of horizontal transfers were the key force to drive the evolution of prokaryotes [1–3], while duplications of genes, partial or entire genomes, and mass gene loss events formed the genotypes of many higher eukaryotes, including higher plants [4–6] and vertebrates [7–10]. The genomic change fixed in generations over time ultimately shapes the biological diversity.

Important information contained in the discrepancies between these evolutions can be extracted and studied with the methods of trees reconciliation. Knowledge of ancestral genomic events provides efficient instruments in a range of fields, like establishing orthology/paralogy relationships between gene families [11–14], functional gene annotations [15–18], reconstruction of ancestral genes and genomes and their dating [19, 20], accurate reconstruction of gene and species trees [18, 21–27], construction of phylogenies based on whole genome data [22, 23], event-based reconstruction of coevolution [28] and its applications in ecology and biogeography [29–31], phylogenetic approaches to predict protein interactions [32], and so forth. A particularly intriguing problem is the* coevolution* of species, genes, and their regulatory systems, including binding sites, protein and RNA factors, DNA and RNA secondary structures, and RNA triplexes, which is poorly understood even in its statement. Further research in this area will shed more light on understanding the principles of concerted evolution at various levels.

In complex studies of coevolution it is vital to develop reconciliation approaches that account for as many various evolution events as possible. Not only inferring the events per se but also their mutual arrangement in time is important. Such an arrangement is called the* evolutionary scenario*. An overview of approaches to define and construct the scenario with trees reconciliation is the scope of this section.

In earlier works, scenarios accounted for gene duplications and losses only [33–36], some later—for only transfers and losses [37–41]. Such incomplete scenarios are useful in certain cases, for example, in studies of the Metazoa where transfers are very scarce, with low-copied or functionally nonredundant gene families or under low rates of duplications and losses [42, 43]. Timing the species tree and, particularly, imposing time scales (slices) are used in recent models to incorporate horizontal transfers [44–46]. The problem of defining and constructing the evolutionary scenario in its broad sense is actively studied, although its full definition is by far not yet obtained.  Section 2 of this paper contains some original results obtained on these problems.

Approaches to substitute the species or even gene trees with a forest or net (graph or hypergraph) and to identify their areas that cannot be described by a tree are important but remain poorly studied [47].

The accuracy of reconciliation methods depends on the quality of initial phylogenetic data, usually gene trees, and multiple alignments in selected cases. The traditional steps of building gene trees (constructing multiple alignments, the choice and configuration of inference methods, robustness verification, etc.) can be nontrivial, especially for the automated generation of phylogenies on genomic scales. These methods are ever developing and are not discussed here. Some approaches are proposed or overviewed, for example, in [48–52], with extensive further referencing provided therein.

To mention is the group of methods that does not rely on gene trees to construct the scenario. Instead, genetic data in extant species of the given species tree is used to reconstruct the same type of data at internal nodes. In [53, 54] the authors addressed the problem of constructing parsimonious scenarios for individual sets of orthologous genes on a fixed species tree. Duplication events are not considered, and a horizontal transfer is not scored separately from an* ab novo* gene gain.

An extensive corpus of studies is devoted to the reconstruction of ancestral molecular characters and properties, rather than inferring discrete evolutionary events on the species tree. Such can be ancestral sequences, their lengths, primary and secondary regulatory structures, the tree areas with potential genetic transfers, and so forth [55–59]. Deterministic and probabilistic models (in particular, the Gibbs field approach) to reconstruct ancestral sequences and secondary structures are discussed in [60–62] presents a dedicated web service. These works remain out of the scope, as do studies of the mutation process and various reconciliation applications. The reader is referred to the original cited works for further details.

### 1.2. Reconciling Gene and Species Trees: The Classic “Embedding *α*” as the Basis of Other Mappings and Mapping Costs

Earlier scenarios accounted only for duplications and losses. Such is the classic definition of mapping *α*, usually referred to as the “embedding *α*.” In [33, 63] it maps vertices of a gene tree into vertices of a species tree. Namely, each vertex *g* of a gene tree *G* is assigned a vertex *α*(*g*) of the species tree *S* that corresponds to the last common ancestor of the species containing the leaves descendants of *g*. Mapping *α* explicitly infers duplications and implicitly losses.

Define edges of tree *S* as* tubes* to distinguish between edges of *S* and *G*. Each root is supplied with an additional* root edge* (or root tube), which ends in a* superroot*; that is, the superroot is the only vertex with the single child. Henceforth, all trees are described as directed downwards from the root.

Consider another definition of the “embedding *α*.” Define mapping *f* as a mapping of vertices in the gene tree *G* into vertices or tubes (often both) in species tree *S* that satisfies the conditions: the leaves in *G* map into leaves in *S* having the same species notations; the superroot of *G* maps into the root tube in *S*; mapping *f* preserves the* natural order* relation on *G* and *S*; which is defined on any tree by the branching order downwards from the root (i.e., this relation keeps the succession of lineages). Additional less determinative conditions are formulated in Sections 2.3 and 2.7 ([45, 64]). In this paper, most definitions are provided in Section 2 and the reader is expected to be acquainted with general terminology used throughout the text.

Definition of *f* continued. The total sum of duplications and losses (the “embedding cost”) has the minimal value on *α* among all costs of possible mappings *f* of gene tree *G* into species tree *S*. The embedding cost of mapping *α* is denoted *c*(*α*); the analogous cost of *f* is denoted *c*(*f*); that is, *c*(*α*) = min⁡⁡{*c*(*f*) | *f*} (where *f* is a variable). In other words, *c*(*α*) and *c*(*f*) are sums of the amounts of gluings and gaps in mappings *α* and *f*, respectively; these numbers can be weighted according to the costs of corresponding event types (in this case, duplications and losses). Thus, mapping *α* can be defined as a global minimum of the embedding cost functional *c*(*G*, *S*, *f*) = *c*(*f*), where variable *f* runs over all mappings of *G* into *S*. Note that the list of event types and the localization of evolutionary events are defined on the species tree individually for each mapping *f* (refer to definitions in Section 2.4).

Algorithmically, mapping *α* is built by induction from leaves toward the root in linear computing time, and its cost is computed simultaneously [65, 66].

Study [45] describes a similar definition of mapping *α*, and a different construction algorithm is applied from the root toward the leaves. It is a useful definition in terms of its extensibility to scenarios with gene horizontal transfers and gains. The presented algorithm simultaneously computes the mapping and its cost.

In [36] all possible reconciliations of gene tree *G* and species tree *S* are considered, that is, all possible mappings *f* of *G* into *S*. This approach is further developed in [43], where *f* maps each vertex in *G* into a vertex or tube in *S*, thus inferring the speciation (if *f*(*g*) is a vertex) and duplication (if *f*(*g*) is a tube) events, respectively. An algorithm described in [43] generates a random reconciliation of *G* and *S*, enumerates all such possible reconciliations, and calculates exactly the minimal number of fixed operations needed to rearrange one reconciliation into the other.

Let only duplications and losses be considered, and let *G* be a binary gene tree with a predefined set of  “reliable” edges. To find is a tree *G*′ with the same set of leaves and containing all clades induced by reliable edges such that *G*′ minimizes the embedding cost of its mapping *α* into a given binary species tree *S*. Algorithms to solve this problem are described in [35, 67]; in [35] the algorithm is proved to find exactly the optimal gene tree *G*′ in cubic time, while [67] offers a heuristic solving algorithm. Similarly, in [68] duplications, losses and transfers are accounted for to find a gene tree *G*′ such that it contains a predefined set of reliable edges (i.e., the induced clades) from *G* and minimizes the embedding cost of any mapping *f* of *G*′ into a given binary species tree *S*. A heuristic solving algorithm is proposed.

An approach to reconcile gene and species trees based on information about synteny of corresponding genes in the genome is proposed in [69]. An algorithm is described to build a forest of trees that reflect the evolution of pairs of neighboring genes by minimizing the embedding cost of gains and losses of the gene pairs. Computing time of this algorithm has the order *n* ^2^ *k* ^2^, where *n* is the number of gene trees and *k* is their maximal size.

### 1.3. The Binarization Problem for Fixed Gene and Species Trees

The algorithm described in [70] has a linear time complexity, and, given a polytomous gene tree *G*, binary species tree *S*, and their mapping *α*, searches for a binarization G* of *G* by first minimizing the total sum of duplications and then the total sum of losses in the obtained set of binarizations.

Study [71] describes a linear time algorithm to binarize the tree *G* against the tree *S* using mapping *α*, provided that only duplications and losses are allowed. A binary resolution *G*′ of a polytomous *G* is constructed such that the resulting binarized gene tree G* optimally reconciles with the species tree *S*; that is, it has the minimal embedding cost compared to other binarizations. Importantly, the algorithm is mathematically proved to find the global minimum of the embedding cost functional *c*(*G*′, *S*, *α*) (*G*′ is a variable). The authors of [71] reference the history of the binarization problem for the case of duplications and losses under fixed *G* and *S*.

Study [25] uses a similar minimization criterion for ∑_*j*_ *c*(*G*_*j*_′, *S*, *f*_*j*_) to binarize many polytomous gene trees *G*_*j*_ against a binary species tree *S* when horizontal gene transfers are allowed, and the variable *f*_*j*_ is an arbitrary mapping (refer to Sections 2.7 and 2.12). In [25] the algorithm is proved to find the globally minimal binarization and possess the complexity determined as follows: if *k* is a maximal degree of polytomy among all vertices in *G*_*j*_, then the computing time has the order of the product of the total number of vertices in initial trees *G*_*j*_ and *S* and coefficient 2^2*k*^.

In [70] it is proved that the optimal binarization problem is NP-complete for the case of a polytomous species tree even if a gene tree is binary. However, heuristics is proposed to handle even nonbinary gene trees. In [72] another heuristic algorithm is proposed to solve the same problem, nevertheless requiring a binary gene tree.

The algorithm described in [73] computes all possible binarizations *S*′ of a polytomous species tree *S* in order to find such S* that minimizes the embedding cost for an input fixed binary gene tree *G* against the variable *S*′. In this search all event types are considered, including transfers; and the variable *f*_*j*_ is an arbitrary mapping. A new condition is imposed: let a vertex *g* in a gene tree be mapped into a vertex *s* in the species tree; then both child clades of *s* contain at least one species from the clade of *g*. The computing time of the algorithm is the product of a polynomial of degree 4 (a function of the number of leaves in the input data) and an exponential functional that depends on the maximal degree of polytomy in the species tree.

Sections 2.12 and 2.13 present an essentially different statement of the binarization problem (refer also to [25]).

### 1.4. Evolutionary Scenarios with Horizontal Transfers: Coevolution of Genes and Their Regulation Systems on a Species Tree

Accounting for gene horizontal transfers in evolutionary models is vital for understanding the evolution of many life forms, especially prokaryotes [1–3]. It also provides efficient tools to study the evolution of molecular systems, establishing orthology/paralogy relationship between gene families [11–14], and so forth. In [74] the authors give a broad view of the perspectives to reconstruct the Tree of Life within the general framework of genome evolution, the role of gene horizontal transfers, duplications and losses in the emergence of new molecular functions, and evolutionary adaptation.

With only duplication events allowed, for a given set of binary gene trees *G*_*j*_ and a binary species tree *S*, consider any mapping *f*_*j*_ of *G*_*j*_ into *S*. In the approach in [75], for each *G*_*j*_ a duplication event *α*(*g*) is attempted closer to the root of a species tree but below *α*(*g*′), where *g*′ is the parent of *g* (if *g* is the root, *α*(*g*) is attempted closer to the root). A functional is proposed that depends on {*f*_*j*_} and equals the sum (over all vertices *s* in *S*) of maximal heights of subtrees in all *G*_*j*_ (not only those that reach the leaves) mapped by *f*_*j*_ into a vertex *s*. The desired are mappings *f*_*j*_ that minimize this functional. A linear complexity proved algorithm to find this global minimum is proposed. Historical references to this approach are provided in [75] and in review [76].

Event-based approaches to study coevolution of various elements are discussed in [28], and their applications in ecology and biogeography are discussed in [29–31]. For example, in [77, and unpublished materials] the authors present a model and an effective algorithm to reconstruct coevolution of genes and their regulatory systems (binding sites, protein and RNA factors, DNA and RNA secondary structures, RNA triplexes, etc.) under horizontal transfers and other events allowed on a species tree. A general coevolutionary scenario was constructed based on a universal functional that combines requirements specific for individual scenarios of the co-evolving elements. Evolutionary events inferred in individual scenarios within the general coevolutionary scenario appear to be biologically consistent (coordinated with each other). Inferring coevolutions is an important and complex problem, which we do not almost discuss in this paper.

### 1.5. Time Slices on the Species Tree as an Approach to Accounting for Horizontal Transfers

When horizontal transfers are included in the model, a gene cannot transfer between two tubes located anywhere on the species tree *S*, a transfer is possible only between the “contemporaries.” To correctly describe transfers, the tree *S* must be partitioned in* time slices*, for example, by dating its tubes or vertices. An approach to do so is presented in [44], where each tube is associated with a time interval, and a transfer between tubes is allowed if their intervals have a non-empty intersection. A corresponding construction algorithm is described in [44], without the complexity assessment. A very complicated original description of the algorithm does not allow us to provide detailed comments.

Assume that to correctly define a transfer in time is to allow it to occur exactly within one time slice (a set of predefined time slices on the species tree *S* is to be fixed). If the correctness condition is not imposed but transfers are allowed, the fastest algorithm constructs a scenario in time of the order *mn*, where *m* and *n* are numbers of leaves in the input gene and species trees [78]. Finding a scenario defined correctly in time is an appealing challenge and requires an intricate imposition of time slices on the tree. Constructing the slices is a difficult problem of its own already at the level of definition. An algorithm with complexity *n* ^3^ that solves it is proposed in [45], albeit without a proper biological justification.

An approach to construct a time-correct scenario is finely elaborated in [45] and, independently, in [46]. The constructing algorithm in [46] uses a prefixed set of time slices and does not consider (similarly to [44]) the common case of a gene transfer with loss of the donor copy. The authors prove the polynomial time complexity of their algorithm, however not providing an exact assessment of the polynomial degree. In [45] the algorithm accounts for all types of transfers and differs conceptually from those proposed in [44, 46]; it is proved to have the complexity of *mh*, where *h* is the number of vertices in a species tree with preimposed time slices and is proved to find the exact global minimum (under certain conditions). The proof is given in [79].

In [80] the following condition (below referred to as the “tofig-condition”) on mapping *f* is formulated. Assume there exists a linear order <_*T*_ at vertices of the species tree *S*, for which: for any tube (*u*, *v*) in *S* the inequality *u*<_*T*_ *v* is valid, and if for two edges, (*u*, *v*) and (*u*′, *v*′), in a gene tree *G* one precedes the other in terms of the natural order on edges, then the upper terminus *a* of the tube that “contains” *f*(*u*) (i.e., *f*(*u*) is this same tube or its lower terminus) must “precede” (in the sense of *a*<_*T*_ *b*) the lower terminus *b* of the tube that contains *f*(*v*′). Under this condition the problem of finding a globally minimal scenario *f* is NP-complete [81, 82]. Strengthening this condition may simplify the situation. For example, let each time slice on *S* consist of tubes equidistant from the root, and let, as mentioned above, horizontal transfers be permitted only within the common slice. Such the condition on *f* implies the tofig-condition if <_*T*_ is a width-first linear order. The problem of finding a globally minimal scenario *f* under the above-mentioned strong condition becomes polynomial in time [45].

The notion of the evolutionary scenario, specifically for a pair 〈gene  tree  *G*, species  tree  *S*〉, is very important in mathematic aspects of the theory of evolution. A realistic scenario is such that accounts for as many different types of gene evolutionary events as possible, including various types of horizontal transfers.

Analogously to mapping *α*, a candidate mapping (scenario) *f* is defined at vertices of the gene tree *G*, with its values being the vertices or tubes (often both) of the species tree *S*, such that *f* keeps the natural orders (the successions of lineages on the trees). Each mapping *f* defines its own set of evolutionary events (exact definitions are provided in Sections 2.3 and 2.7). As in mapping *α*, each event type is assigned a cost. Analogously to the embedding cost *c*(*α*) of mapping *α*, the cost *c*(*f*) of a candidate mapping *f* is the sum of event costs defined by *f*, which may be weighted according to the reliability of corresponding vertices and the type of event. The problem is to find the mapping *β* (scenario *β*) that globally minimizes the total cost *c*(*f*) under certain constrains, which almost always need to be imposed on its design.

The cost of the pair 〈*G*, *S*〉 is the cost of its minimal scenario *β* and is denoted *c*(*G*, *S*). Therefore, one needs to minimize the functional *c*(*G*, *S*, *f*) over all mappings *f* of *G* into *S* to obtain the desired scenario *β* and the value *c*(*G*, *S*).

An alternative approach is to describe the scenario in terms of a stochastic process on a species tree. Selected relevant approaches are proposed in [25].

An algorithm to construct a scenario *β* for a gene tree *G* and a species tree *S*_0_ derived by partitioning *S* in time slices, is described in [25, 45, 79]. The running time of the algorithm has the order of the product of *m* and the number of leaves in *S*_0_. The algorithm, its proof, and all definitions from [45, 79] are reproduced in [83], where the algorithm was extensively tested on novel data. In [84] the same (as the authors perceive) algorithm is applied to different biological data.

The importance of taking into account suboptimal scenarios that can become optimal under slight variations of the costs of event types is demonstrated in [80]. An approach to deal with suboptimal scenarios is proposed in [25], where the authors also examine the case of gene gain using the outgroup approach (refer to the extended event list in Table  1 in [25]).

### 1.6. Constructing the Supertree

The definitions and algorithms of trees reconciliation and construction of the scenario (mapping) stated above can be applied to another long studied problem: given a set {*G*_*j*_} of gene trees, find the tree S*, for which the* total cost *∑_*j*_ *c*(*G*_*j*_, *S*, *f*_*j*_) of all events for pairs 〈*G*_*j*_, S*〉 reaches the global minimum (*S* and *f*_*j*_ are variables; usually *f*_*j*_ = *α*_*j*_, which gives the cost ∑_*j*_ *c*(*G*_*j*_, *S*)). The tree S* is called a* supertree*. In this statement, the supertree may be imposed certain constraints depending on the initial gene tree data that need to be taken into account when optimizing the total cost functional.

In the classic sense, the supertree is constructed with no constraints by merging input trees using a variety of heuristic methods based on various tree compatibility criteria. In distance approaches, the supertree is found by minimizing the average distance between it and all input trees. Defining a proper distance is therefore of importance. In the framework of trees reconciliation, this problem reduces to the minimization of the functional defined via the total cost of evolutionary events for trees *G*_*j*_ over all *j*. The classic and still commonly used distance was introduced in [33] as the total cost of duplications and losses (and transfers, if allowed).

In some approaches, the supertree construction step is preceded by filtering out leaves, subtrees, or entire gene trees that do not satisfy certain reliability conditions [85]. The discarded elements can later be used to detect areas of “active” evolution on the supertree. We do not discuss such kind of approaches here.

The problem of building the supertree is NP-complete if* no constraints* are imposed on the desired tree S*, even when only duplications and losses are allowed [86]. This stimulated the development of heuristic methods and attempts to reformulate the problem itself.

*Heuristic Approaches.* Among such is the quartet method that consists of two phases. At the first phase, trees are built for all quartets of species; here the choice of the reliability function to assess quartet topologies plays the important role; refer, for example, to [87]. At the second phase, the supertree is built by optimally reconciling the quartet species trees using a heuristics. In different implementations of the second phase, the supertree is constructed either “from root to leaves” [88] or “from leaves to root” [89]. The method produces an unrooted tree.

Rooted supertrees are produced by the triplet method, an analog of the quartet method, where the final tree is obtained by assembling triplet trees also using heuristics, for example, as described in Phase 2 of the supertree building algorithm from [25]; refer to Section 2.10 below.

Other methods use heuristics to maximize the functional of clades matching among two trees (rooted supertrees are produced) [90] or use a matrix representation of multiple trees [91]. A simple method to root species trees is proposed in [25, Suppl. 1].

Out of the scope of this paper remain other approaches to infer a species tree, such as the supermatrix strategies, which are popularly used in many phylogenetic studies of particular groups as well as larger taxa. In the supermatrix design, sets of orthologous genes sampled across the compared species are aligned, concatenated into a “superalignment” (supermatrix) and processed for computing one tree. In so doing, this method combines partially overlapping species samplings in the input orthologous sets to accommodate all species in one tree. Although the supermatrix approach relies on the well-established methodology of inferring gene trees, there exist many pitfalls that limit its application to larger analyses on a genomic scale. Among them are the strict requirement on orthology, missing data in sparse supermatrices, and different modes of evolution exhibited by different supermatrix partitions (often exacerbated by disparities in their size) and even by individual positions in the alignments, which requires the usage of sophisticated evolutionary models and causes inevitable computational burden that may become intractable with larger datasets [92–95].

In this context, fine selection of orthologs has received much attention as a problem of high relevance and arduous both ideologically and computationally. Approaches to this problem diverge into reconciliation-based (e.g., [11–14]) and graph clustering methods (e.g., [96–100]). The authors in [100] proposed a quadratic in time complexity clustering algorithm to construct orthologous protein families based on sequence similarity (and local synteny in certain cases). It was applied to mitochondrial, plastid, and some other (unpublished) genomic data. The obtained clusters well conform with known protein functional annotations, independently constructed orthologous groups, and other protein characteristics. The clustering revealed some lineage-specific proteins. Thus, mitochondria of the vine* Vitis vinifera* were found to encode proteins also typical for plastids, which implies that a horizontal genetic flow between these organelles had happened in the past [100]. 

*Reformulation of the Problem*. The development of novel reconciliation approaches and their effective solving algorithms with low (polynomial) complexity that are mathematically proved to find the global minimum (presumably the correct supertree) holds a good perspective. The algorithm originally developed by the authors [23, 64] introduces a condition that allows to effectively find the global minimum of the total cost functional. The condition constrains the desired supertree S* to contain only clades from the input gene trees and certain combinations of them. Under this condition and if only duplications and losses are allowed, the algorithm is mathematically proved to find the global minimum of the cost functional in time cubic of the input data size [64]. Solving the same problem for the case of transfers is an important perspective. This approach is based on a different principle compared to other known methods.

### 1.7. Probabilistic Definitions of the Evolutionary Scenario: Evolution as a Stochastic Process and Coalescent Approaches

The definition of the clade probability as a fraction of trees containing a given clade was introduced in [101]. The authors argue that the correct supertree commonly contains all clades from the initial tree set with the probability >1/3.

The species tree reconstruction under the assumption of numerous transfers is discussed in [102]. Using a probabilistic approach, it is shown that the species phylogeny is tree-like even with a high transfers content, that is, when their number linearly depends on the average number of leaves per tree. Conversely, in [24] it is mathematically proved that the triplet method recovers the correct supertree with high probability only if transfers are not many. Studies [24, 102] well reference this approach.

A stochastic procedure to construct a scenario with all types of events, including transfers, is proposed in [25]. The authors describe an algorithm to compute expectation values of the event numbers in each tube and over all tubes of the species tree. The proposed approach can also be used to determine other characteristics of the process.

In the first subsection below we briefly overview two groups of publications operating with quite sophisticated probabilistic approaches that need to be further discussed in terms of the probability theory. The second subsection is devoted to the coalescent theory.

#### 1.7.1. Evolution as a Stochastic Process

A type of stochastic processes other than in [25] is considered in [103]. Fix a gene tree *G* and a species tree *S*, with tube lengths corresponding to times; paths from the root to each leaf have equal lengths. An oracle is fixed that assigns to each natural number *n* and tube *d* the probability of the outcome “*d* contains exactly *n* duplications.” Here, a mapping *f* of vertices in *G* into vertices and tubes in *S* is defined under the condition: if for any child *g*_1_ of *g* the inequality *α*(*g*) ≠ *α*(*g*_1_) is valid, then *α*(*g*) = *f*(*g*) or *f*(*g*) is a tube having *α*(*g*) as its lower terminus.

A probability of *f* is the probability of tube *d* to contain exactly |{*x* ∈ *G* | *f*(*x*) = *d*}| duplications multiplied over all *d* in *S*. Recall that the sign |·| stands for the number of the set elements, the cardinality of the set. To find are (i) the highest likelihood among all possible mappings *f* (ii) the mapping f* with the highest likelihood itself, and (iii) the numbers of duplications in each tube *d* under the mapping f*. The authors describe a polynomial of degree 5 heuristic algorithm for (i) and exponential complexity algorithms that find exact solutions for each of the three tasks.

The following statement is considered in [104]. Fix *G* and *S* (the root tube *d*_0_ is located upwards the root in *S*) with tube lengths corresponding to times and *λ* and *μ* being the intensities of duplications and losses, respectively. The intensities are constant across all tubes and are parameters of a linear death-birth process (its formal definition is provided at the end of this subsection).

For each vertex *g* in *G* denote *A*(*g*) as the set *A*(*g*) = {*f*(*g*) | *f*}; that is, *A*(*g*) contains vertices and tubes *f*(*g*) from *S* for a variable mapping *f* and a fixed argument *g*. Call mappings *f* and *h* adjacent if the following conditions are valid: *f* and *h* differ at exactly one vertex *g*, *f*(*g*) and *h*(*g*) are comparable in *S* (in terms of the natural order on tree *S*), and there exist no elements from *A*(*g*) strictly in-between them.

In [104] a tree *G*′ is defined and generated by the below stochastic process; *G*′ is then compared with the initial tree *G*. Let us first informally describe the stochastic process for *G*′. The root tube of *S* contains the start of gene lineages that descend downwards and bifurcate at each vertex of *S* (the divergence events). In each tube, each gene lineage undergoes duplications or losses with given intensities *λ* and *μ*. In case of a loss of the lineage terminates, in case of a duplication, it bifurcates into two descendent lineages in this tube. All lineages terminate in leaves, and only then the process ends to generate a tree *G*′ (inside the tubes) and its natural mapping into *S*; all lineages terminated before leaves are discarded and not included in *G*′. The tree *G*′ and its natural mapping into tree *S* are generated in any realization of this random process from the root toward leaves in *S*. More precisely, arrange slices by ascending order when the current total amount of lineages changes by 1. Let the root part of the tree *G*′ be generated at instant *t*. If at that instant the number of lineages in a tube increases by 1, a lineage is chosen equiprobably in the tube and bifurcated; if it decreases by 1, this lineage terminates.

The probability *P*(*G*, *f*) of mapping *f* is the probability that the random process generates a tree *G*′ isomorphic to the tree *G* through mapping *f*. The probability *P*(*G*) of tree *G* is the sum of probabilities of all its mappings in *S*;* a conditional probability P*(*f* | *G*) is defined as *P*(*G*, *f*)/*P*(*G*). By substituting *P*(*G*) in the denominator with a sum over a given subset of mappings (defined *K*), we obtain the definition of a *K*-*approximated* conditional probability and denote it *P*_*K*_(*f* | *G*).

Define a graph, where all vertices are mappings *f* of *G* into *S* and edges connect adjacent vertices. Fix an arbitrary spanning tree *T* in the graph that is rooted by mapping *α*; and let *K* be a connected subgraph with *k* vertices in *T*. In [104] the authors prove the following: for all mappings *f* from *K*, the probability *P*(*f* | *G*) is computed with the time and memory of *O*(|*G*|^2^|*S*| + *k*(|*S*| + |*G*|)) and the *K*-approximation *P*_*K*_(*f* | *G*)—with the time and memory of *O*(|*S*||*G*| + *k*(|*S*| + |*G*|)).

Experiments with biological data were performed to obtain realistic values of intensities *λ* and *μ* of duplications and losses.

A *d*-probability is the sum of conditional probabilities *P*(*f* | *G*) of all mappings *f* from *T*, which are separated from the root *α* by maximum *d* edges; such mappings are called *d*-mappings. Computer simulations showed that, (i) with the increase of *d* (from 0), the *d*-probability soon reaches the plateau and (ii) for each mapping *f* before the plateau, the value *P*_*K*_(*f* | *G*) approximates *P*(*f* | *G*) with high accuracy if *K* includes all mappings before the plateau.

An algorithm realizing the approach of [104] was developed and applied to biological data in [105]. Earlier related results are in [104, 105].

Probabilistic modeling of gene evolution can also be applied to model sequence divergence, as described in [106] and, with more detail, in [107]. A model and an algorithm are proposed in [107] to simultaneously infer gene trees, the species tree, and expectations of duplications and losses in each tube of the species tree, given a set of multiple alignments. Further relevant references are provided in [107]. 

*A Formal Description of the above Described Process*. Let *P* be a linear death-birth process applied to the tree *S*. The process argument is time *τ* taking on a value from 0 to *τ*_0_, where *τ*_0_ is the path length between the root and a leaf. At each vertex *s* define time *t*(*s*) as the length of the path from the vertex to the root; tube *d* = (*s*_1_, *s*_2_) (*s*_1_ closer to the root) “contains the instant” *τ* if *t*(*s*_1_) ≤ *τ* ≤ *t*(*s*_2_). The value of *P*(*τ*) is a set of pairs: a tube *d* possessing instant *τ* and the number *d*(*τ*) of gene lineages in the tube at instant *τ*. The definition of *d*(*τ*) is as follows: *P*(0) is a set consisting of the single pair 〈root  tube  *d*_0_, 1〉; that is, *d*_0_(0) = 1; let for a nonroot tube *d* = (*s*_1_, *s*_2_) hold *τ* = *t*(*s*_1_); then, by induction from root to leaves assume that *d*(*τ*) equals *d*′(*τ*), where *d*′ is the parent tube for *d*; determine the change of *d*(*τ*) in a small time interval *δt* of the argument such that *d* contains *τ* + *δt*. For each tube *d* = (*s*_1_, *s*_2_) possessing instant *τ*, define conditional (transition) probabilities of the number *d*(*τ* + *δt*) of gene lineages at the instant *τ* + *δt* if at the previous value *n* = *d*(*τ*) is known:

(1) $$\begin{array}{c} \text{Pr}\left\{d\left(\tau +\delta t\right)=n+1\;|\;d\left(\tau \right)=n\right\}=n\lambda \delta t+o\left(\delta t\right), \end{array}$$

where *λ* is duplications intensity, and

(2) $$\begin{array}{c} \text{Pr}\left\{d\left(\tau +\delta t\right)=n-1\;|\;d\left(\tau \right)=n\right\}=n\mu \delta t+o\left(\delta t\right), \end{array}$$

where *μ* is losses intensity,

(3) $$\begin{array}{c} \text{Pr}\left\{\left|d\left(\tau +\delta t\right)-n\right|>1\;|\;d\left(\tau \right)=n\right\}=o\left(\delta t\right). \end{array}$$

The end of process definition.

#### 1.7.2. Coalescent Approaches

In this group of studies, modeling the evolution of genes along a species tree includes a novel approach and an evolutionary event of novel type, the incomplete lineage sorting (refer to [108, 109] for the theory and references provided therein). Below we go with some detail into this important concept, which is grounded on the mathematical theory of the reverse time. On trees, the “direct time” refers to the time directed from the root to the leaves, and the “reverse time” reverses this direction.

The problem of reversing time in stochastic processes was first visited by Kolmogorov [110]. Kingman [111] had found that the probability distribution on phylogenies in large populations is described by a special type of random processes named coalescence and analyzed them in reverse time using the earlier model of population evolution proposed by Wright [112] and Fisher [113]. These works laid the foundation of the coalescence theory, and Kingman gave it further development and formulated it for continuous time.

*The Central Idea of the Model in Short*. Consider the sets of “parents” and “children,” *G*_*n*_ and *G*_*n*+1_, at *n*th and (*n* + 1)th generations, each consisting of *N* elements. Assume that a multivalued mapping *D*_*n*_ of *G*_*n*_ into *G*_*n*+1_ is surjective and satisfies the condition: the values of any two different elements from *G*_*n*_ are disjoint subsets of *G*_*n*+1_. Such the mapping *D*_*n*_ is an inverse of the single-valued mapping *F*_*n*_ of *G*_*n*+1_ into *G*_*n*_ (informally, children are mapped into their parent). There is exactly *N* ^*N*^ different mappings *F*_*n*_, which are equiprobable, and thus each *F*_*n*_ has the probability *N* ^−*N*^. It is also assumed that for all generations *G*_*n*_ all *F*_*n*_ are independent random maps.

This simple description is equivalent to conventional definitions of the Wright-Fisher-Kingman model, which we describe below for the comparison.

The jth individual (“parent”) from *G*_*n*_ produces *ν*_*j*_ individuals (“children”) in generation *G*_*n*+1_ and dies; *ν*_*j*_ are supposed to be mutually independent (over *j*) and follow the Poisson distribution:

(4) $$\begin{array}{c} P\left\{\nu_{j}=r\right\}=e^{-\lambda }\frac{\lambda^{r}}{r!}. \end{array}$$

Let a population contain *N* individuals at each generation *G*_*n*_; that is, the condition ∑_*j*_ *ν*_*j*_ = *N* must be imposed to fix the population size over generations. The joint distribution of *ν*_*j*_ is multinomial:

(5) P{νj=rj,1≤j≤N}=∏j(e−λ·(λrj/rj!))e−Nλ·((Nλ)N/N!)=(Nr1,…,rN)NN=N!r1!⋯rN!NN,

where ∑_*j*_ *r*_*j*_ = *N*.

The map *F*_*n*_ can be interpreted as the random choice of a parent from *G*_*n*_ by each individual from *G*_*n*+1_; this choice is equiprobable. The latter formula defines the probability of the event “the first parent has *r*_1_ children, the next parent has *r*_2_ children, and so on down to *r*_*N*_”.

The evolution in reverse time is a transition from *G*_*n*+1_ to *G*_*n*_ and deeper toward the root. The probability of any two individuals from *G*_*n*+1_ having different parents is (1 − (1/*N*)); and having different parents in *s* preceding generations (down to *G*_*n*−*s*+1_) is (1−(1/*N*))^*s*^. The probability of *k* fixed different individuals from *G*_*n*_ having *k* different parents in one preceding generation is

(6) $$\begin{array}{c} P_{k}=\left(1-\frac{1}{N}\right)\left(1-\frac{2}{N}\right)\cdots \left(1-\frac{k-1}{N}\right), \end{array}$$

and having *k* different parents in *s* preceding generations (down to *G*_*n*−*s*_) is *P*_*k*_^*s*^. Consider a limit of *P*_*k*_^*s*^ with variable *s* and *N* → *∞*. The lifetime of one generation is assumed to be Δ*t* = 1/*N*; that is, in time *t* = *s* · Δ*t* = *s*/*N*, one observes *s* generations, *s* = *Nt*. The probability of *k* fixed individuals having *k* different parents (in the limit under *N* → *∞*) over fixed time *t* (the lifetime of [*Nt*] generations) is

(7) lim⁡N→∞Pk[Nt]=lim⁡N→∞(1−k(k−1)2·1N)Nt=exp⁡(−k(k−1)2t).

A simple interpretation of the last formula: *k* individuals can form *k*(*k* − 1)/2 pairs, the probability that any pair of individuals over time *t* does not share a common parent is exp⁡(−*t*). In random time *t* (with the parameter *k*) a random pair of individuals is chosen and assigned a parent. Time *t* is defined by the random variable *T* distributed exponentially as *P*{*T* > *t*} = exp⁡(−(*k*(*k* − 1)/2)*t*). The choice of the pair is equiprobable because the probability of choosing a pair from *k*(*k* − 1)/2 possible pairs is (*k*(*k* − 1)/2)^−1^. An unpaired individual is a parent of itself. The obtained parents are further paired with each other analogously until no further pairing is possible.

This process can be described as building a phylogenetic tree for given *k* individuals (leaves). In a next (inductive) tree level in the direction root ward, a pair is chosen from *m* current parent individuals and coupled under a new common node to form two new edges with the same length *t* equal to the value of random variable *T* distributed exponentially with the parameter *m* (at the start of induction *m* = *k*):

(8) $$\begin{array}{c} P\left\{T>t\right\}=\exp\left(-\frac{m\left(m-1\right)}{2}t\right). \end{array}$$

An unpaired individual is projected on the next level and forms a new vertex and a new edge of length *t* connecting the two vertices. Thus, an unpaired individual is parental to itself.

This process is called the* coalescence* and ends with building a rooted tree with *k* leaves.

Other coalescences are considered in [109, 114]. Namely, fix *k* individuals at a time instant, with *k*_1_ mutants and *k*_2_ wild type, *k*_1_ + *k*_2_ = *k*. Assume that all mutants evolve from one parent that acquired a single mutation and all its descendants are mutants. Denote *A*, |*A*| = *k*_1_ the extant population of mutants and by  *B*, |*B*| = *k*_2_ the population of extant wild types. The genealogy of *A* bearing the mutation is a subtree in the genealogy of the whole population, the union of *A* and *B*. The coalescence process is used to find a phylogenetic tree such that lineages of *A* coalesce earlier than any lineage from *A* forms a common parent with any lineage from *B*. A coalescence that satisfies this constraint is called* conditional *[114]. Algorithmically, the coalescence tree building process is running multiple times until the described tree containing the clade *A* is built.

Another constraint imposed on the tree building process is studied in detail in [109]. Namely, consider a species tree *S* with lengths (times) given for all tubes such that all paths from any node to the leaves are equal, thus defining the age of the node. Conditional coalescence can be applied to build a gene tree *G* along with its mapping into *S*, that is, the evolution of the gene inside the species tree [109]. At the start of induction, each gene leaf is assigned to its corresponding species leaf in the tree *S*. In* reverse* time, in the resulting tree any two gene leaves existing in two different species tubes form the common gene parent of at least the age of the common parent of the corresponding species. Thus, gene lineages may coalesce much later than their containing species. Such an event is called the* incomplete lineage sorting*.

The described process is applied to the case when the mutant is replaced with a duplicated copy of a gene that acquired a mutation after the duplication [108] had occurred. If a part of a population undergoes genetic change, it may result in the formation of subspecies. After the speciation event, the change is usually fixed in this subspecies. The duplicated copy of a gene survives, in contrast with the models like mapping *α* that operate with species as discrete units.

Study [108] also introduces the* interim locus tree* concept based on conditional coalescence. A gene tree is mapped into the interim locus tree, which then maps into the species tree. The species tree evolves in direct time, from root to leaves. However certain ideas in the description of this approach remain hard to understand.

## 2. Constructing the Evolutionary Scenario and the Supertree: Algorithms and Proofs

This section (Sections 2.1–2.13) of the article describes the original solutions and corresponding mathematical proofs proposed by K. Y. Gorbunov and V. A. Lyubetsky for the two problems in the field of trees reconciliation: inferring gene evolution along a species tree and trees reconciliation into a single tree (including the case of polytomous trees). These developments apply to a diverse and important subject of the evolution of species, genes, and their regulatory systems considered in concert or separately.

### 2.1. Statement of Two Problems

Studies [23, 25, 45, 64, 79] tackle two important and sophisticated problems in bioinformatics. The obtained results are partially reviewed in Section 1 of the paper, which also provides an extended biological background and relevant references.

*The first problem* is to reconstruct a gene evolution along a species tree or, in other words, to construct a mapping of a gene tree into a species tree and to build the scenario.* The second problem* is to reconcile a set of gene trees into one common species tree. A specific facet of the second problem is to build a supertree (by globally minimizing a suitable functional commonly referred to as the “cost”) for the given set of trees. This problem is extended to the hard case of polytomous data, especially polytomous input trees.

In the above-mentioned works [23, 25, 45, 64, 79] only concise formulations are provided, while in this section we give mathematical statements and proofs to describe the two problems on the case when only gene duplication, loss, and divergence during speciation are the considered evolutionary events. Following on, we describe in detail the extension of the developed algorithms to incorporate other types of gene evolution events and/or the case of polytomous gene trees.

The first problem is solved in polynomial (often linear, at maximum cubic) time even for the case of incorporating time slices and horizontal gene transfers. In Section 2.7 it is proved that the corresponding original algorithm of cubic time complexity finds exactly the global minimum; that is, the model is exactly solvable.

In its traditional statement, the second problem cannot be solved algorithmically in polynomial time, as it is proved to be NP-complete. Known exponential solutions (based on various enumerators) are computationally too intensive, and do not guarantee that the optimal solution (the global minimum of a functional) is found if heuristics are applied to stop the search. Moreover, the accuracy of approximating the global minimum by a heuristic solution is not clear at all.

Complete proofs are first given for the case of no time slices and gene transfers. The discussion of the second problem follows next. A solving algorithm cubic of the initial data size is suggested that finds the exact* conditional* (refer to Section 2.5) global minimum under no gene transfers; that is, in this case the model is also exactly solvable. However, for the case of transfers the algorithm is not mathematically proved to find the exact conditional global minimum, which remains an important open problem. The heuristic solution for this case and its usage are described in [23, 25, 45, 64, 79].

We end this section with giving a solid mathematical background for the second problem for a fixed set of polytomous rooted gene trees. This problem is also discussed in [25].

### 2.2. Auxiliary Definitions

Let a gene tree *G* and a species tree *S* be given. The trees are rooted and binary, and oriented downwards from the roots. Recall that edges of the tree *S* are referred to as* tubes* to distinguish between the edges of *S* and *G*. Each root is supplied with an additional* root edge* (or root tube), which initiates in a* superroot* and ends in the root; that is, the superroot is the only vertex inducing the single child. Each leaf is labeled with a species name. Species names in *S* are unique; species names in *G* may duplicate if it contains several genes from the same species (paralogous genes). Species names in *G* are a subset of those in *S*. A* subtree* is a part of a tree that consists of a vertex, an edge entering the vertex from above (the subtree root edge), and all vertices and edges descending downwards. A* clade* of a subtree is a set of species names present in all its leaves; a clade of a vertex is the clade of its subtree. For a clade *V*, the corresponding tree is referred to as a* tree over V*.* A paralogous subtree* (with respect to a species) in *G* is such a maximal subtree that has all leaves marked with one species (i.e., its clade is a singleton; the paralogs are in-paralogs for this species). Pruning of a subtree *T* from tree *G* is a deletion of all edges and vertices in *G* belonging to *T* followed by merging of the two edges, incoming in and outcoming from the upper terminus of the root edge in *T*. A* child* of a vertex is another vertex located directly downwards, that is, at a distance of one edge. Remember that ≥ and > mean the natural order on any tree as defined in Section 1.2. The* natural order* relation is defined analogously on a set of edges (tubes), a set of vertices, or a united set of edges and vertices. The terms “lower” and “upper” refer to the natural order of the tree branching downwards from the root. 

Let *e*_+_ be the lower terminus of edge *e*, and let *e* ^+^ be its upper terminus.

### 2.3. Definition of Mapping with Duplications and Losses Only: Reconciling Gene and Species Trees

A* mapping* *f* of a gene tree *G* into a species tree *S* is an assignment of each vertex in *G* to a vertex or tube in *S*, the superroot is mapped into the root tube, and each leaf is mapped into a leaf with the same species name. Two conditions are imposed on *f*: if a vertex is mapped into a tube, its child is mapped into the same tube or downwards (lower); if *f*(*g*) is a vertex in *S*, then for children *g*_1_ and *g*_2_ of *g*, the values *f*(*g*_1_) and *f*(*g*_2_) are in the two different descendent (lower) subtrees of *f*(*g*) in *S*.

Examples and illustrations of mappings are given in [23, 25, 45, 64].

### 2.4. Definitions of Gene Duplication and Loss and Their Localization on the Species Tree

Let mapping *f* be fixed. In *f*, a* duplication *is a nonsuperroot vertex *g* for which *f*(*g*) is a tube, a* divergence* is a nonleaf vertex *g* for which *f*(*g*) is a vertex, and a* loss* is a pair 〈*e*, *s*〉 of edge *e* in *G* and vertex *s* in *S* such that, for the upper terminus *e* ^+^ and the lower terminus *e* ^+^ of *e*, we observe *f*(*e*_+_) < *s* < *f*(*e* ^+^). If the clade of a child of *s* contains no species from *G*, the loss is called* implicit* (as it is induced by species in *S* but not in *G*). Otherwise, the loss is called* explicit*. A duplication is* located* in the corresponding tube in *S*, a divergence in the corresponding vertex in *S*, and a loss in vertex *s*.

Each event type (duplication, loss, divergence, etc.) is assigned a nonnegative cost value. A* cost* of mapping *f*of *G* into *S* is the sum of event costs inferred in this mapping. A* cost* of mapping {*f*_*j*_} of a set of gene trees *G*_*j*_ into a species tree *S* is the total cost of mappings *f*_*j*_ of *G*_*j*_ into *S*. Denote these costs *c*(*G*, *S*, *f*) and *c*({*G*_*j*_}, *S*, {*f*_*j*_}) = ∑_*j*_ *c*(*G*_*j*_, *S*, *f*_*j*_), respectively. The variables *f* and {*f*_*j*_} are often implied but not written explicitly.

A mapping with the minimal cost is called* canonic* and designated *α*, [33, 63]. A linear algorithm to construct it is described in [65, 66]; more details can be found in [20].

*DenoteV*_0_  a set of all species names in all given gene trees *G*_*j*_.

### 2.5. Formulating the Problems of Reconciling Two (Gene and Species) and Many (Gene) Trees

During the reconciliation of two trees, for given gene *G* and species *S* trees, a mapping *f* is sought for such that it globally minimizes the functional *c*(*G*, *S*, *f*) over the variable *f*.

During the reconciliation of many trees, for a given set {*G*_*j*_} of gene trees, a set of mappings {*f*_*j*_} and a tree S* are sought for such that they globally minimize the functional *c*({*G*_*j*_}, *S*, {*f*_*j*_}) over the variables {*f*_*j*_} and *S*. This minimization is done under the ad hoc* condition*: each *S* must contain only clades belonging to a* predefined* set *P* of subsets of set *V*_0_; all clades from {*G*_*j*_} are by default already contained in *P*.

Traditionally, the second problem requires the* unconditional* (absolute) minimization. We refer to the introduced reformulation as to the* parametric* (over the parameter *P*) or* conditional* minimization (optimization).

### 2.6. The First Problem under Gene Duplications and Losses Only: Reconciling Gene and Species Trees

If *g* is not the superroot vertex *g*_0_ in tree *G*, denote LCA(*g*) the last common ancestor in *S* of a clade defined in *G* by a subtree with the root vertex *g*. A* second definition* of canonic mapping *α* slightly differs from the definition provided in Section 1.2 as follows. Let *α*(*g*_0_) = *d*_0_, where *d*_0_ is the root tube. If for both children *g*_1_ of vertex *g* holds the inequality LCA(*g*) ≠ LCA(*g*_1_), then *α*(*g*) = LCA(*g*); otherwise *α*(*g*) is a tube incoming to LCA(*g*) from the upwards. Informally, *α*(*g*) may be visualized as located “inside the tube.” Hereafter, only the second definition of canonic mapping *α* is used. Analogously, a set {*f*_*j*_} of mappings *f*_*j*_ is canonic if each *f*_*j*_ (of *G*_*j*_ into *S*) is canonic. From the remark to Lemma 2 it follows that the second definition of *α* and its definition based on the global cost minimization are equivalent. The second definition is given in [33, 63].

**Lemma 1**

If mapping *f* is not canonic *α*, then for each vertex *g* the inequality *f*(*g*) ≥ *α*(*g*) is valid, and, at least for one *g*, *f*(*g*) > *α*(*g*).

**Proof**

Clade *f*(*g*) contains clade *g*, that is proved with induction from leaves to *g*. The first inequality follows from the statement above and the observation: if *α*(*g*) is a tube, then *f*(*g*) is not its lower terminus, as the terminus already contains a descendant of *g*.By definition, *f* cannot map two comparable vertices to one. The condition *f* ≠ *α* implies the last statement of the lemma.

**Lemma 2**

For any mapping *f* different from *α*, the amount of duplications for *f* is not less than for *α*, and the amount of losses is strictly greater for *f* than for *α*.

**Proof**

Consider a duplication for *α*; that is, *α*(*g*) = *d*, where *d* is a tube. Then *f*(*g*) cannot be a vertex *s* > *d*, as then, by definition of mapping, one of the children of *g* must map in a descendent subtree of *s* not containing *d*. It is impossible, as the clade *g* does not intersect with the clade of the descendent subtree (it is further referred to as the* bifurcation effect*). According to Lemma 1, *f*(*g*) ≥ *d*, therefore *f*(*g*) is a tube. Consequently, a duplication for *α* remains a duplication for *f*. Note that a divergence for *α* may become a duplication for *f* ≠ *α*.

First prove that the amount of losses is not less for *f* than for *α*. Consider a loss (*e*, *s*) for *α*. If *f*(*e*_+_) < *s*, it remains a loss for *f*, because, according to Lemma 1, *f*(*e* ^+^) ≥ *α*(*e* ^+^) > *s*. The equality *f*(*e*_+_) = *s* is false due to the bifurcation effect.

Next, due to *f*(*e*_+_) ≥ *α*(*e*_+_) obtain that *f*(*e*_+_) is comparable with *s*. If *f*(*e*_+_) > *s*, the loss (*e*, *s*) corresponds to at least two losses, (*e*_1_, *s*) and (*e*_2_, *s*), in *s* for *f*, where *e*_1_ ≠ *e*_2_ and both *e*_1_, *e*_2_ < *e*. Indeed, on any path from *e*_+_ downwards, an edge will induce a loss in *s* (a divergence cannot occur on the path due to the bifurcation effect). If (*e*′, *s*′) is another loss for *α*, it corresponds to two losses in *f* differing by *s* or *e*, given that *e*′ is incomparable with *e*. Thus, there exists a multivalued injective mapping that maps each loss in *α* to one or two losses in *f*, with nonintersecting images. Since for a fixed vertex *s* the property of being an explicit or implicit loss in *s* depends on the tree *G* only, and losses for *f* are of the same type (explicit or implicit) as for *α*.

*The Last Statement of the Lemma*. By the condition and Lemma 1, there exists a vertex *g* in *G*, for which *f*(*g*) > *α*(*g*). The two cases are possible: (i) *f*(*g*) and *α*(*g*) are tubes, (ii) *f*(*g*) is a tube, and *α*(*g*) is a vertex. Indeed, for a vertex *f*(*g*) a contradiction arises according to the bifurcation effect.

*Case (i).* Let *s* be an arbitrary vertex, for which *f*(*g*) > *s* > *α*(*g*). Consider two nonoverlapping paths from *g* to the leaves. On both paths there occur edges *e*_1_ and *e*_2_ inducing for *f* losses *l*_1_ and *l*_2_ in *s* (the bifurcation effect). As *s* > *α*(*g*), the paths from *e*_1_ and *e*_2_ to the root contain either none or one coincident loss in *s* for *α*. Consequently, the losses *l*_1_ and *l*_2_ either are not contained in the mapping image *μ* or constitute the image of one coincident loss. In both alternatives, the amount of losses is greater for *f* than for *α*.

*Case (ii).* Consider an arbitrary path from *g* to a leaf. According to the bifurcation effect, it contains an edge *e* such that (*e*, *α*(*g*)) is a loss for *f*. This loss is not contained in the mapping image *μ*, as there exists no edge *e*′ on the path from *e* to the root such that (*e*′, *α*(*g*)) is a loss for *α*.

**Remark 3**

Let *f* and *h* be two different mappings. If for any vertex *g* holds the inequality *f*(*g*) ≥ *h*(*g*), then by substituting *α* to *h* in Lemma 2 we prove that the amounts of duplications for *f* is not less than for *h*, and the amount of losses is greater for *f* than for *h*. An analogous statement is proved in [115] for vertex-to-vertex mapping functions.

Henceforth,* assume* that the cost of a divergence is less than the cost of a duplication; this condition is likely to be biologically justified. Then, by Lemma 2, a canonic mapping *α* is a solution of the first problem, that is, the two definitions of *α* coincide. Further, if in a set {*f*_*j*_} a mapping *f*_*j*_  is not canonic, then its replacement with a canonic mapping will reduce the total cost. Thus, in the second problem the only true variable is the desired species tree *S*.

Lemmas 1-2 solve the first problem only for the case when the gene duplication, loss, and divergence during speciation are considered. Lemmas 4–7 prove certain properties of *α* and will be used in the proofs of Theorems 8-9.

**Lemma 4**

If a gene tree *G* is obtained from a species tree *S* by pruning some subtrees from *S*, then for a canonic mapping *α* of *G* into *S* duplications and explicit losses are absent, and each pruned subtree (with the root tube *d*) induces an implicit loss in *d* ^+^. Conversely, if for a canonic *α* there are no duplications, then *G* is obtained from *S* by pruning some subtrees.

**Proof**

Prove the lack of duplications with induction on the amount of pruned subtrees. At the start of induction, *G* = *S*, and only a divergence event is possible.

An induction step from *G*_*n*_ to *G*_*n*+1_, where *n* is the number of pruned subtrees. If in *G*_*n*_ it is true that *α*(*g*) is a vertex *s* and a vertex *g* is not pruned, then both clades of its children in *G*_*n*_ and *G*_*n*+1_ are subsets of the clades of corresponding children of vertex *s*. These children in *G*_*n*+1_ still map in *α* strictly below *s*. Therefore, in *G*_*n*+1_ also *α*(*g*) = *s*, vertices in *G*_*n*+1_ map into vertices, and a duplication does not occur.

Prove the absence of explicit losses by contradiction. An induction step. Let a vertex *s* contain an explicit loss 〈*e*, *s*〉 after pruning a (*n* + 1)th subtree *T*. Then in tree *S* both clades of the children of *s* contain species from *G*_*n*+1_. Consequently, there exists a vertex *g*, for which *α*(*g*) = *s*, as such *g* existed in *G*_0_ and was not pruned. Thus, edge *e* does not exist.

A vertex of tree *S*, a former image of the upper terminus of the root edge of a pruned subtree, contains an implicit loss in *G*_*n*+1_ induced by a new edge in the tree *G*_*n*+1_ formed after merging of two initial edges.

Let us prove that, for any vertex *s* in *S*, a set *M*_*s*_ = {*g* | *α*(*g*) ∈ *T*_*s*_}, where *T*_*s*_ is a subtree rooted in *s*, defines a tree obtained from *T*_*s*_ by pruning certain subtrees. If *s* is a root, this statement is obtained. Prove it with induction. If *s* is a leaf, the statement is obvious. An induction step. Assume a vertex *g*, for which *α*(*g*) = *s*. Then the sought subtrees set is the union of the corresponding sets for children *s*_1_ and *s*_2_ of vertex *s*. Assume there is none such *g*. Then the images of members of *M*_*s*_ belong to one child subtree of vertex *s* (put it the child *s*_1_); otherwise *s* will contain the last common ancestor of members of *M*_*s*_. The sought set of subtrees consists of a subtree rooted in *s*_2_ and the set of subtrees for *s*_1_.

**Lemma 5**

In a canonic *α* of *G* into *S*, each leaf tube terminating in species *s* contains the number of duplications equal to the number of nonleaf vertices in a paralogous subtrees for *s* in the gene tree *G*.

**Proof**

Denote the number of nonleaf vertices in Lemma 5 by Par(*G*, *s*). Any internal vertex of a paralogous subtrees induces a duplication according to the definition of *α*. And, conversely, such a duplication corresponds to a vertex in *G* that is contained in a paralogous subtree for *s*.

**Lemma 6**

Fix a gene tree *G* over a subset of  *V*_0_. Let species trees *S*_1_ and *S*_2_ be both defined over *V*_0_, each containing a certain subtree *S*. Then the two canonic mappings of *G* into *S*_1_ and *G* into *S*_2_ produce the same set of events in *S*; that is, the set of events in a subtree does not depend on the subtree's complement (the rest of the tree).

**Proof**

Let *V* be a clade of the subtree *S*. Vertices mapped in *S* coincide in both mappings, as their clades belong to *V*; the image of such vertices coincides in both mappings, as it depends only on *S*. By definition of the duplication, the set of duplications in *S* coincides in both mappings. Let 〈*e*, *s*〉 be a loss in one mapping *α*, where *s* is a vertex in *S*. Then in another mapping *α* the image of edge *e*_+_ remains constant, and the image of *e* ^+^ also remains constant (if belongs to *S*) or remains external to *S* (if does not belong to *S*) above the image of *e*_+_ and, therefore, above *s*. In both cases, 〈*e*, *s*〉 is a loss also in another mapping *α*. Thus, the set of losses also coincides between two mappings *α*.

**Lemma 7**

Fix a gene tree *G* over a subset of *V*_0_. Let *V* be a subset of *V*_0_, and a species tree *S*_1_ over *V*_0_ contains a subtree *T*_1_ over *V*. Let a tree *S*_2_ be derived from *S*_1_ by substituting the subtree *T*_1_ with a subtree *T*_2_ over *V*. Then the canonic mappings of *G* into *S*_1_ and *G* into *S*_2_ produce the same set of events in the complements to the subtrees *T*_1_ and *T*_2_; that is, the set of events in a complement to a subtree does not depend on this subtree.

**Proof**

By definition of mapping *α*, vertices mapped outside *S*_*i*_ are the same for *i* = 1,2, as their clades do not belong to *V*, or, equivalently, their LCA images are not contained in *S*_*i*_. Each such vertex *g* has the same *α*-image. Indeed, the values of LCA(*g*) coincide on *S*_1_ and *S*_2_; that is, if on one of the *S*_*i*_ the *α*(*g*) is a tube, it is a tube on the other. By definition of a duplication, the set of duplications outside *S*_*i*_ coincides between the two mappings. Let 〈*e*, *s*〉 be a loss in one mapping, where *s* does not belong to *S*_*i*_. Then in the other mapping the image of *e* ^+^ does not change, and that of *e*_+_ either does not change (if not belong to *S*_*i*_) or remains in *S*_*i*_ (if belongs to *S*_*i*_) and thus is below *s*. In both cases, 〈*e*, *s*〉 is a loss also in the other mapping. Consequently, the set of losses also coincides between the two mappings.

### 2.7. The First Problem under Gene Duplications, Losses, and Horizontal Transfers with Imposed Time Slices: An Algorithm to Reconcile Gene and Species Trees (Building an Evolutionary Scenario)

The generalization of mapping *α* to incorporate gene transfers has long been a daunting task. Here we describe an original approach to solve it.

Let the species tree *S* impose certain time slices; refer to Sections 1.4-1.5; the slices are ranked from the root to leaves. The slices must satisfy the single condition: if *d*_1_ ≤ *d*_2_, then the rank of *d*_1_ is not less than the rank of *d*_2_. For example, a *k*th slice contains all tubes distanced by the amount of *k* tubes from the root; in [25] the slices are constructed with an additional condition: all leaf tubes belong to one slice. The latter condition is inessential in further definitions and is accepted without discussion.* Denote* *d*_1_ ~ *d*_2_ for tubes *d*_1_ and *d*_2_ if *d*_1_ ≠ *d*_2_ and *d*_1_, *d*_2_ belong to the same time slice.

With horizontal transfers, we formulate a similar (refer to Sections 2.3, 2.6) but inductive definition of* mapping f* of a gene tree *G* into a species tree *S* and its* cost* [64]. Simultaneously with *f*, an additional tree *G*′ is defined as derived from *G* by inserting* new vertices with a single child*. The number *n* of new vertices on an edge defines the number of transfers: if *n* is even, a gene (more precisely, edge *e* in *G*; see below) underwent *n*/2 transfers without retention of the gene donor copy, and if *n* is odd, a single transfer with and (*n* − 1)/2 transfers without retention.

Let *e* be any edge in *G*,and let *d* be any tube in *S*. The definition of *f* and *G*′ is based on an important auxiliary definition of the* inner tree* and its* cost* for* any pair*  〈*e*, *d*〉. All pairs of the form 〈edge  from  *G*, tube  from  *S*〉 are partially ordered: a pair 〈*e*, *d*〉 is lower than 〈*e*′, *d*′〉 if *e* < *e*′ or *e* = *e*′ and the rank of tube *d* is greater than that of *d*′. Pairs 〈*e*, *d*〉 are visited from leaves to the root in the linear order consistent with the described partial order. Remember that any vertex is identified by its incoming edge.

#### 2.7.1. Defining the Inner Tree for the Pair 〈*e*, *d*〉

The start of induction. Let *e* and *d* be any leaf edge and any leaf tube, respectively, and let *d*′ be a tube with the species of gene *e* in its lower terminus. If *d* ≠ *d*′, the inner tree contains the pair 〈*e*, *d*〉 and its single child, the pair 〈*e*, *d*′〉; this corresponds to a transfer without retention of the donor copy from *d* into *d*′. If *d* = *d*′, the inner tree consists of the single pair 〈*e*, *d*〉. The* cost* of this tree is the cost of a transfer without retention if *d* ≠ *d*′ and is zero otherwise (for more details on transfers refer to Sections 1.4-1.5) [23, 25, 45, 64, 79].

Thus, the inner tree is a marked tree; the mark of a vertex has the form 〈*e*, *d*〉.

#### 2.7.2. An Induction Step

Let *e* and *d* be a nonleaf edge and tube, respectively. Then the* inner tree* and its* cost* for the pair 〈*e*, *d*〉 are defined as follows depending on the sequential choices listed below. Namely, for any 〈*e*, *d*〉, the outcome is selected according to rules 1–6 below, with some of the rules describing a choice. In square brackets is the description of applicability. Otherwise said, a set of inner trees is defined, with each inner tree describing an alternative evolution of gene *e* inside species *d*.

(1) [Tube *d* has the single child *d*_1_]. The inner tree consists of the pair 〈*e*, *d*〉 with the single child 〈*e*, *d*_1_〉 that roots the already known inner tree for 〈*e*, *d*_1_〉. This tree has the cost equal to that of 〈*e*, *d*_1_〉. Descriptively, lineage *e* enters the next tube.

(2) [Tube *d* has two children, *d*_1_ and *d*_2_]. The inner tree consists of the pair 〈*e*, *d*〉 with the single child 〈*e*, *d*_1_〉 that roots the inner tree for 〈*e*, *d*_1_〉 or the child 〈*e*, *d*_2_〉 that roots the inner tree for 〈*e*, *d*_2_〉 (only one case must be chosen). The cost of this tree is the cost of the chosen 〈*e*, *d*_*i*_〉 plus the cost of a loss (explicit if the other child *d*_*j*_ possesses at least one leaf from *G* and implicit otherwise). Descriptively, lineage *e* survives only in one of the two tubes.

(3) [Edge *e* has children *e*_1_ and *e*_2_]. The inner tree consists of the pair 〈*e*, *d*〉 with two children, 〈*e*_1_, *d*〉 and 〈*e*_2_, *d*〉, which root the inner trees for pairs 〈*e*_1_, *d*〉 and 〈*e*_2_, *d*〉. Its cost is the sum of costs of trees 〈*e*_1_, *d*〉 and 〈*e*_2_, *d*〉 and a duplication. Descriptively, lineage *e* is duplicated in *d*.

(4) [Edge *e* has children *e*_1_ and *e*_2_; tube *d* has children *d*_1_ and *d*_2_]. The inner tree consists of the pair 〈*e*, *d*〉 with two children, 〈*e*_1_, *d*_1_〉 and 〈*e*_2_, *d*_2_〉, which root the inner trees for pairs 〈*e*_1_, *d*_1_〉 and 〈*e*_2_, *d*_2_〉. Its cost is the sum of costs of trees 〈*e*_1_, *d*_1_〉, 〈*e*_2_, *d*_2_〉, and a divergence. In the alternative choice, *e*_1_ and *e*_2_ swap. Descriptively, lineage *e* diverges in *d*.

(5) [Edge *e* has children *e*_1_ and *e*_2_]. The inner tree consists of the pair 〈*e*, *d*〉 with two children, 〈*e*_2_, *d*′〉 and 〈*e*_1_, *d*〉, which root the trees for pairs 〈*e*_2_, *d*′〉 and 〈*e*_1_, *d*〉, where *d*′ ~ *d*. Its cost is the sum of costs of the trees for 〈*e*_1_, *d*〉, 〈*e*_2_, *d*′〉, and a transfer with retention. In the alternative choice, *e*_1_ and *e*_2_ swap. Descriptively, lineage *e* duplicates in *d* with a subsequent transfer into *d*′ and retention of the donor copy in *d*.

In rule 6 the definition of *d*′ is used in the same sense.

(6) In this rule, descriptively, lineage *e* duplicates in *d* with subsequent transfers into *d*′ and losses of the donor copy in *d*.

(6.1) [Tube *d*′ has the single child *d*_1_′]. The inner tree consists of the pair 〈*e*, *d*〉 with the single child 〈*e*, *d*′〉, which also produced the single child 〈*e*, *d*_1_′〉 that roots the tree for 〈*e*, *d*_1_′〉. The cost of this tree is the sum of costs of 〈*e*, *d*_1_′〉 and a transfer without retention. Descriptively, lineage *e* enters from *d*′ into the next tube *d*_1_′.

(6.2) [Tube *d*′ has two children, *d*_1_′ and *d*_2_′]. The inner tree consists of the pair 〈*e*, *d*〉 with the single child 〈*e*, *d*′〉, which also produces the single child 〈*e*, *d*_1_′〉 that roots the tree for 〈*e*, *d*_1_′〉. The cost of the tree is the sum of costs: 〈*e*, *d*_1_′〉, a transfer without retention, and a loss in *d*_2_′ (explicit if *d*_2_′ possesses at least one leaf from *G* and implicit otherwise). The alternative is the choice for 〈*e*, *d*_2_′〉. Descriptively, lineage *e* survives only in one of the two tubes.

(6.3) [Edge *e* has children *e*_1_ and *e*_2_]. The inner tree consists of the pair 〈*e*, *d*〉 with the single child 〈*e*, *d*′〉, which produces two children, 〈*e*_1_, *d*′〉 and 〈*e*_2_, *d*′〉, which root the trees for 〈*e*_1_, *d*′〉 and 〈*e*_2_, *d*′〉. The cost of the tree is the sum of costs of 〈*e*_1_, *d*′〉, 〈*e*_2_, *d*′〉, a transfer without retention, and a duplication in *d*′. Descriptively, lineage *e* duplicates in *d*′.

(6.4) [Edge *e* has children *e*_1_ and *e*_2_; tube *d*′ has children *d*_1_′ and *d*_2_′]. The inner tree consists of the pair 〈*e*, *d*〉 with the single child 〈*e*, *d*′〉 that produces two children, 〈*e*_1_, *d*_1_′〉 and 〈*e*_2_, *d*_2_′〉, which root the trees for 〈*e*_1_, *d*_1_′〉 and 〈*e*_2_, *d*_2_′〉. The cost of this tree is the sum of costs: 〈*e*_1_, *d*_1_′〉, 〈*e*_2_, *d*_2_′〉, a transfer without retention, and a divergence. In the alternative choice, *e*_1_ and *e*_2_ swap. Descriptively, lineage *e* transfers in *d*′ and then diverges in the lower terminus of *d*′.

(6.5) [Edge *e* has children *e*_1_ and *e*_2_]. The inner tree consists of the pair 〈*e*, *d*〉 with the single child 〈*e*, *d*′〉 that produces two children, 〈*e*_2_, *d*′′〉 and 〈*e*_1_, *d*′〉, which root the trees for 〈*e*_2_, *d*′′〉 and 〈*e*_1_, *d*′〉, where *d*′′ ~ *d*′ ~ *d* (tube *d*′′ differs from tubes *d* and *d*′). The cost of the tree is the sum of costs of 〈*e*_1_, *d*′〉, 〈*e*_2_, *d*′′〉, and transfers with and without retention. Descriptively, lineage *e* transfers in *d*′, duplicates in *d*′ with a subsequent transfer into *d*′′ and retention of the donor copy in *d*′. The end of the inner tree definition.

Remember the notation: subscript and superscript indices of “+” designate lower and upper termini, respectively, or edges and tubes; *e*_0_ and *d*_0_ are the root edges in trees *G* and *S*.

The inner tree *T* for the pair 〈*e*_0_, *d*_0_〉 is used to construct a candidate mapping *f* = *f*_*T*,〈*e*_0_,*d*_0_〉_ and simultaneously a candidate tree *G*′, which vertices are mapped into vertices and tubes of tree *S*. Namely, when running the vertices of an inner tree *T* for the pair 〈*e*_0_, *d*_0_〉 from its leaves upwards to the root consider the following. Let *e*_1_ and *e*_2_ be children of edge *e*, and let *d*_1_, *d*_2_ be children of tube *d*. In square brackets is the description of applicability followed by the rule formulation. Each pair 〈*e*, *d*〉 marks the corresponding vertex in tree *T*:

(0) *f*(*e*_0_^+^) = *d*_0_;

(1) [leaf vertex 〈*e*, *d*〉] *f*(*e*_+_) = *d*_+_;

(2) [vertex 〈*e*, *d*〉 has a child of the form 〈*e*_*i*_, *d*〉] *f*(*e*_+_) = *d*. If the other child has the form 〈*e*_*j*_, *d*′〉, a new vertex *g*′ (with the single child) is inserted on edge *e*_*j*_ in current *G*′ and *f*(*g*′) = *d*′. If the edge *e*_*j*_ already received a number of single-child vertices, a new single-child vertex is inserted in the edge upwards of the already received;

(3) [〈*e*, *d*〉 has the children 〈*e*_1_, *d*_1_〉 and 〈*e*_2_, *d*_2_〉] *f*(*e*_+_) = *d*_+_;

(4) [〈*e*, *d*〉 has the single child 〈*e*, *d*′〉]. Insert two vertices *g*′ and *g*′′ on edge *e* in current *G*′(each with the single child; *g*′ is higher than *g*′′) and *f*(*g*′) = *d*, *f*(*g*′′) = *d*′.

The set of candidate* mappings f* of *G*′ into *S* is obtained. Candidate* partial mappings f*_*T*,〈*e*,*d*〉_ for any pair 〈*e*, *d*〉 are obtained analogously, as well as candidate partial trees *G*_*T*,〈*e*,*d*〉_′. The end of the candidate mapping definition.

*A scenario* (*mapping*) f* is a candidate mapping that minimizes the total cost of its evolutionary events.

The role of the inner tree for 〈*e*_0_, *d*_0_〉 is to describe the evolution of a gene described by a tree *G* inside the species described by a tree *S*; if a pair 〈*e*, *d*〉 is a vertex of the inner tree then edge *e* evolves inside tube *d* at least along its certain segment.

An algorithm to build the scenario trivially repeats the same induction that was used to define the inner tree: for every pair 〈*e*, *d*〉, the choice will minimize the cost over all possible choices. The same induction is used to build the mapping that coincides with canonic *α* when transfers are not considered.

To account for gene gain events, we introduce an auxiliary outgroup, a tube d* connecting the root of *S* with an auxiliary outgroup species d*. Introducing time slices generates tubes on the outgroup tube with single children, which we also denote d*. Gene lineage that evolves into the outgroup tube and later transfers back into the initial species tree *S* is considered as* gained*. The start of induction is modified as follows: for d*, the cost of a transfer without retention is replaced with a fixed gain cost. Induction steps are also modified. In rules 2-3, the costs of loss and duplication are zeroed for *d*_0_ and d*, respectively. Rule 3′ is added: for a pair 〈*e*, *d*_0_〉, the inner tree consists of 〈*e*, *d*_0_〉 with two children, 〈*e*_1_, *d*_0_〉 and 〈*e*_2_, d*〉, which root the inner trees for pairs 〈*e*_1_, *d*_0_〉 and 〈*e*_2_, d*〉, where *e*_1_ and *e*_2_ are children of *e*, and d* is the upper outgroup tube. The cost of this tree is the sum of costs for 〈*e*_1_, *d*_0_〉 and 〈*e*_2_, d*〉. In the alternative choice, *e*_1_ and *e*_2_ swap. In rule 4, the cost of a divergence is zero for *d*_0_. A condition is added in rules 5, 6.1, 6.2, 6.3, 6.4, and 6.5: tubes *d*′, *d*′′ are not in the outgroup; for d*, the cost of a transfer with retention (rule 5) or without retention (rule 6) is replaced by the gain cost.

In [25] we describe an even more extended list of evolutionary events. The nontrivial definitions and algorithm above were proposed and thoroughly tested in [45]. In [79] the* complexity of the algorithm* was mathematically proved to be* cubic* with respect to the number of vertices in the species tree that contains time slices. In [45] it was mathematically proved that the algorithm finds the minimal mapping and its cost under the presence of horizontal transfers.

The first problem is solved for the general case.

### 2.8. The Second Problem: Phase 1 of the Supertree Building Algorithm under Gene Duplications and Losses Only

Hereafter, all mappings are canonic *α*. Only duplication, loss, and divergence events are considered.

Consider a set of gene trees {*G*_*j*_} with a set of species called  *V*_0_. To find is a species tree S* over *V*_0_, for which the total cost of individual tree mappings is globally minimal. It is an NP-complete problem. To overcome this limitation, we reformulate the problem of unconstrained optimization into a biologically justified constrained (conditional) optimization problem. Constrain the solution space to contain only species trees *S* satisfying the condition: all clades of *S* belong to a predefined set *P*, which includes at least all clades of input gene trees. Thus, S* must also satisfy this condition. The parameter *P* is nontrivial and is introduced to overcome the NP-complete nature of the problem. A “true” species tree may not exist in this solution space, depending on the degree of consistency of the input set of clades.

The proposed original algorithm of solving the second problem consists of two phases. An exact solution is obtained during Phase 1, provided that the conditional optimization problem is solved under a certain condition.

If the condition is not valid, a follow-up heuristic procedure implemented in Phase 2 can be invoked, which outcome depends on the data generated during Phase 1. As with real data the existence of the unconstrained solution in the solution space for a fixed *P* is usually unknown, one can either empirically expand the set *P* or take the heuristic solution obtained during Phase 2. In computer simulations the latter strategy produced better results (data not shown).

Description of Phase 1. Standard approaches are used to define algorithmic relations over sets from *P*: the “inclusion of one set into another,” “intersection of two sets is empty,” and “cardinality of a set.” Also, the algorithmic relation is defined between vertices of *G*_*j*_ (separately for each *j*) and their clades from *P*. Different vertices (even within one tree) may correspond to the same clade; the set *P* may contain sets that do not correspond to any clade in the input gene trees.

For each set *V* from *P* the set of all its partitions is defined. A partition is a pair 〈*V*_1_, *V*_2_〉 of nonempty nonintersecting subsets *V*_1_, *V*_2_ of set *V* that belong to *P* and their union equals *V*; partitions are easily calculated by verifying the condition |*V*_1_| + |*V*_2_| = |*V*|. Sets from *P* that can be so partitioned down to singletons are defined as* basic*; all singletons are also defined as* basic*. The set *P* may contain nonbasic sets. Thus, an initial *V*_0_ may be nonbasic, which invokes Phase 2 of the algorithm. By induction, we enumerate all basic sets according to the increasing of their cardinality. For each basic set, Phase 1 constructs a tree *S*(*V*) over *V*, called a * basic tree*, and computes its* cost*. In the algorithm implementation, the construction of basic trees and computing their costs are naturally combined. For any singleton *s* from *P*, tree *S*(*s*) contains the single leaf (the root) *s* and the root tube; its* cost* is zero if there are no paralogous trees for *s* and is the cost of one duplication multiplied by ∑_*j*_Par(*G*_*j*_, *s*) otherwise (refer to Lemma 5).

#### 2.8.1. Definition of Basic Trees *S*(*V*) and Their Costs: The Induction Step

Fix nonsingleton basic set *V* from *P* and enumerate all its partitions into basic sets *V*_1_ and *V*_2_ with lesser cardinality.

For each partition, compute a* new cost c*(*V*, *V*_1_, *V*_2_) as follows. Denote *V*(*g*) the clade of a vertex *g*. Let *g*_1_ and *g*_2_ be children of *g*; if *g* is a superroot, then *g*_1_ = *g*_2_. Run each *g* in all *G*_*j*_ and compute the following numbers *q*_1_, *q*_2_, *q*_3_′, and *q*_3_′′.

The number *q*_1_ of vertices *g* in all *G*_*j*_, for which *V*(*g*_1_)⊆*V*_1_ and *V*(*g*_2_)⊆*V*_2_; (or otherwise: *V*(*g*_1_)⊆*V*_2_ and *V*(*g*_2_)⊆*V*_1_ (the sign ⊆ stands for “a subset”)); the number *q*_2_ of vertices *g* in all *G*_*j*_, for which *V*(*g*) is a subset of *V* and at least one of the sets *V*(*g*_1_) or *V*(*g*_2_) has non-empty intersection both with *V*_1_ and with *V*_2_.

Select gene trees *G*_*j*_ for which (i) the root clade intersects with both sets *V*_1_ and *V*_2_ and (ii) the root clade intersects with one of the sets and not with the other.

Compute the number *q*_3_′ of edges *e* = (*e* ^+^, *e*_+_) in all *G*_*j*_ satisfying (i) and the new condition (iii): *V*(*e*_+_) is a subset of *V*_1_ or *V*_2_, and either *e* ^+^ is the superroot, or for the child *g* ≠ *e*_+_ of *e* ^+^, the set *V*(*g*) is a subset neither of *V*_1_ nor of  *V*_2_. Also compute the number *q*_3_′′ of edges in all *G*_*j*_ satisfying (ii) and (iii).

Define a new cost

(9) c(V,V1,V2)=c(V1)+c(V2)+cdiv⁡·q1+cdup·q2+clos1·q3′+clos2·q3′′,

where *c*_div⁡_ is the cost of a divergence, *c*_dup_ is the cost a duplication, *c*_los1_ is the cost of an explicit loss, and *c*_los2_ is the cost of an implicit loss.

Assume that *c*(*V*, *V*_1_*, *V*_2_*) is the minimal cost among *c*(*V*, *V*_1_, *V*_2_) for all partitions 〈*V*_1_, *V*_2_〉 of *V*. The tree *S*(*V*) is* obtained* by merging trees *S*(*V*_1_*) and *S*(*V*_2_*) under the join root, where 〈*V*_1_*, *V*_2_*〉 is one of the pairs satisfying the minimal cost requirement. The* cost* of *S*(*V*) is defined as *c*(*V*, *V*_1_*, *V*_2_*).

Phase 1 outputs a set {*S*(*V*) | *V*} of basic trees *S*(*V*) for each basic set *V*. The end of Phase 1.

### 2.9. Justification of Phase 1

Let *S*_1_ be an arbitrary species tree over *V*_0_ that includes a subtree *S*(*V*).* Denotec*(*V*) the total cost of events in *S*(*V*) in canonic mappings of all gene trees *G*_*j*_ in *S*. The cost *c*(*V*) differs from the total cost *c*({*G*_*j*_}, *S*_1_) as it accounts only for the events in *S*(*V*); of course, if *V* = *V*_0_, the costs are equal. If any tree *S*_2_ over *V*_0_ is considered that contains *S*(*V*) as a subtree, the cost *c*(*V*) will remain the same as for *S*_1_ according to Lemma 6. Thus, the cost *c*(*V*) is a function of the tree *S*(*V*) and does not depend on its comprising tree *S*_1_.

Evidently, if the second conditional problem is solvable, then *V*_0_ is a basic set, and the tree *S*(*V*_0_) is the solution according to Theorem 8.

**Theorem 8**

A basic tree *S*(*V*) globally minimizes the functional *c*(*V*) in the conditional problem for *V* if the problem is solvable. The algorithm constructs *S*(*V*_0_) in time |*P*|^3^ + |*P*|^2^ · |*V*_0_| · *n*, where *n* is the number of input trees *G*_*j*_.

**Proof**

Obviously, the solution exists if and only if *V* is a basic set. The time complexity is proved in [64].

By induction, enumerate basic sets according to the increasing of their cardinality. For a singleton set, the statement of Theorem 8 follows from Lemma 5. Let *V* be a nonsingleton set. Prove that, for each partition of *V* into *V*_1_ and *V*_2_, the computed value *c*(*V*, *V*_1_, *V*_2_) equals the sum of event costs in a tree *T*, where *T* is a result of merging trees *S*(*V*_1_) and *S*(*V*_2_) under the common root (as mentioned above, the value *c*(*T*) depends on *T* only). Denote *r* the common root, and *d*—the tube entering the root (the root tube). There are three groups of considered events: (i) events in *S*(*V*_1_), (ii) events in *S*(*V*_2_), and (iii) events occurring in *r* or in *d*. By inductive assumption, the total event cost of groups (i) and (ii) is *c*(*V*_1_) + *c*(*V*_2_).

Examine the total event cost of group (iii). From definitions of mapping *α* and the events, it easily follows that

(1) *α*(*g*) = *r* (a divergence event) if and only if the condition on *g* corresponding to the number *q*_1_ in the algorithm description is satisfied;

(2) *α*(*g*) = *d* (a duplication event) if and only if the condition on *g* corresponding to the number *q*_2_ in the algorithm description is satisfied;

(3) pair 〈*e*, *r*〉 is a loss if and only if condition (iii) in the algorithm description is satisfied; the loss is explicit if condition (i) on *G*_*j*_ is satisfied and implicit if condition (ii) is satisfied.

Thus, the algorithm finds the numbers of duplications in *d*, divergences in *r*, explicit and implicit losses in *r*, and their total cost. Consequently, the value *c*(*V*, *V*_1_, *V*_2_) is computed correctly.

Let a certain tree *T*(*V*) be the global minimum of the functional *c*(*V*) = *c*(*T*(*V*)) if all its clades belong to the set *P*. The root bifurcation corresponds to a partition of *V* into two basic sets, *V*_1_ and *V*_2_. If subtrees *T*(*V*_1_) and *T*(*V*_2_) are replaced with trees *S*(*V*_1_) and *S*(*V*_2_), respectively, then by Lemma 7 the functional *c*(*V*) does not decrease (indeed, if, e.g., *T*(*V*_1_) is replaced by *S*(*V*_1_), the cost of the events from group (i) does not decrease, and the total cost of groups (ii) and (iii) remains constant). Consequently, such a replacement does not affect the global minimum, and trees over *V*_1_ and *V*_2_ in the desired solution can be legitimately considered those *S*(*V*_1_) and *S*(*V*_2_) that are already constructed at previous steps of the algorithm. The algorithm will output as *S*(*V*) the global minimum of the functional *c*(*V*).

#### 2.9.1. Remark

According to Lemma 5, the cost *c*(*V*) includes the total cost of duplications in all paralogous subtrees over all *G*_*j*_ over all species from *V*. Therefore, the costs of singletons can be any constants, as the optimal tree *S*(*V*) does not depend on them. The set {*G*_*j*_} can also be simplified by replacing all paralogous subtrees with singleton subtrees.

Phase 1 of the algorithm produces a set {*S*(*V*) | *V*} of basic trees, where *V* runs over all basic sets. If the set *V*_0_ of all species is not basic, it will not contain a tree over *V*_0_. In this case, Phase 1 returns no conditional supertree; that is, the conditional problem has no solution.

A natural question is “how to determine if the degree of consistency of the input set of trees suffices for the correct supertree to exist?” An empiric directive for the moment can be that the trees are consistent enough if *V*_0_ is a basic set.

### 2.10. The Second Problem: Phase 2 of the Supertree Building Algorithm

The set *P* is not unambiguously defined by the initial set of gene trees *G*_*j*_. For this reason, a heuristics is implemented in Phase 2 of the algorithm to solve the unconditional problem and assemble basic trees *S*(*V*) into one species tree S* over *V*_0_ under a certain fixed *P*. This heuristic solution largely depends on the outcome of Phase 1. The assembling can be done using a variety of known methods. We propose an original* ad hoc* “augmentation” method described below.

Consider a tree *S* over a set *V*⊆*V*_0_. Its* cost c*(*S*) is defined as the total cost of mappings of* all basic trees* (with two or more leaves) pruned to contain only species from the set *V*.

Let *V* contain only three species. The* basic cost c*(*V*) is the minimal cost *c*(*S*) among all trees *S* over *V*. The* subbasic cost c*′(*V*) is the minimal cost *c*(*S*) strictly greater than *c*(*V*). The* reliability R*(*V*) is defined as (*c*′ − *c*)/*c*′. By enumerating all such *V*, find a tree *S* over *V* with a nonzero reliability and the minimal value of *c*(*V*)·(2 − *R*(*V*)). If for any *V* the cost *c*′(*V*) does not exist, the algorithm terminates. The final tree *S* is the result of the basis of the induction.

An inductive step is similar. Let a tree *S* with *n* ≥ 3 species be obtained. Consider all pairs: species *s* from *V*_0_ not contained in *S* and edge *d* from *S* including its root edge. The edge *d* is broken in two by inserting a new vertex connected with a newly added leaf *s*, thus generating a new tree *S*′. The basic cost *c*(*s*) is the minimal cost *c*(*S*′) when *s* is fixed and *d* is a variable. The subbasic cost *c*′(*s*) is the minimal cost *c*(*S*′) strictly greater than *c*(*s*). The reliability *R*(*s*) is defined as above. By enumerating all *s* find a tree *S*′ with a nonzero reliability and for which *c*(*s*)·(2 − *R*(*s*)) is minimal. If *c*′ does not exist for a species *s*, the species is marked as unreliable and not used in Phase 2. An augmentation step is a transition from *S* to *S*′; the steps are continued until the current *S*′ contains all successfully attempted species from *V*_0_. The resulting species tree is the output of Phase 2 of the algorithm.

The correctness of Phase 2 is proved by Theorem 9. Informally, the topologies of trees *G*_*j*_ in Theorem 9 are assumed to share at least some topological similarity.

**Theorem 9**

Let the cost of an implicit loss be zero. If there exists a tree *S*′ over *V*_0_ such that each basic tree *S*(*V*) can be obtained by pruning *S*′ to contain only species from *V*, then the augmentation leads to a species tree with the zero cost, and the conditional problem is solved. The converse statement is also true.

**Proof**

In the first statement additionally, intermediate trees also have zero costs.

If a tree *T* over *V* is obtained by pruning the tree *S*, then all basic trees pruned to *V* are also prunings of *T*, and, by Lemma 4, the tree *T* has the zero cost. Thus, the augmentation, in where all trees are prunings of *S*, is the desired process. Obviously, such the process exists. The converse statement follows from Lemma 4.

### 2.11. Modification of Phase 1

If topologies of the initial trees *G*_*j*_ strongly contradict (an example is provided in [64]), then Phase 2 produces a tree with a nonzero cost; that is, according to Theorem 9, there exists a basic tree that cannot be obtained by pruning the output of Phase 2 to contain only species from the set *V*. This situation occurs because the basic trees are optimal in terms of the functional *c*(*V*), not in terms of the more accurate total mapping cost.

Computer simulations suggest (data not shown) that Phase 2 performs more accurately in the below case. Let *V* be a fixed subset of *V*_0_ and an element of *P*. Prune each initial gene tree *G*_*j*_ to *V* (denote the result *T*_*j*_ : *V*) and each element *A* from *P* to *V*(denote the result *A* : *V* = *A*∩*V*; *P* : *V* = {*A* : *V* | *A*runs  over*P*}). For a fixed *V*, apply Phase 1 to the sets {*T*_*j*_ : *V* | *j*} and *P* : *V*. Let *T*(*V*) be a basic tree over *V*, if such exists. Apply Phase 2 to the set {*T*(*V*) | *V*runs  over*P*} and* denote* the result S**. An analog of Theorem 9 is easily proved for S** with Lemma 10 stated below. If a set *V* is basic for 〈{*G*_*j*_}, *P*〉 and 〈{*T*_*j*_ : *V* | *j*}, *P* : *V*〉, the basic trees over *V* may be different.

**Lemma 10**

If a set *V* is basic for 〈{*G*_*j*_}, *P*〉, then it is basic for 〈{*T*_*j*_ : *V* | *j*}, *P* : *V*〉.

**Proof**

Since *V* belongs to *P*, it also belongs to *P* : *V*. Use induction on the increase of |*V*|. Singleton sets are always basic. If *V* is a nonpruned basic set, it can be partitioned into two nonpruned basic subsets. By inductive assumption, the subsets are pruned basic. Then *V* is also pruned basic.

The running time of modified Phase 1 is obviously |*P*| times greater compared to standard Phase 1. For both versions of Phase 1, the complexity of Phase 2 has the order of |*P* | ·|*V*_0_|^5^, which is proved in [25].

### 2.12. Definitions of Binarization and Paralogous Binarization

Hereafter, only a canonic mapping *α* is considered and applied to polytomous trees (in the definition of *α* “for both children” is naturally replaced with the “for all children”, refer to Section 2.6). Fix a polytomous gene tree *G*. Describe the procedure that starts from the initial *G* and iteratively derives *G*′. Let in this procedure a tree *G*′ be already derived and possess a polytomous vertex *g*. Then arbitrarily divide the children of *g* with their incoming edges into two nonempty parts *A* and *B*, and for each part (with the corresponding subtrees) introduce an intercalating edge connecting a new vertex (the ancestor of this part) with *g*; if a part is a singleton, the corresponding new vertex is eliminated (none of the trees contains edges with one child). The tree *G*′ so acquires two or one new vertices and keeps the ones inherited from *G*, and the vertex *g* becomes binary. The described operation is the* step of binarization* of vertex *g* against partition (*A*, *B*). Repeat the operation until all polytomous vertices are found. Name the obtained “resolved” tree *G*′ a* candidate binarization* of *G*.

Fix a binary species tree *S* and the polytomous gene tree *G*. Among all candidate binarizations *G*′ of *G*, find such *G* ^#^ = *G* ^#^(*S*) that has the minimal embedding cost among the values *c*(*G*′, *S*, *α*) (*G*′ is a variable); name *G* ^#^ a* binarization *of *G against S*.

By definition, for given *G* and *S*, an edge *e* from *G enters* (downwards) a tube *d* in *S* if

(10) $$\begin{array}{c} \alpha \left(e^{+}\right)\;\geq d^{+}\;>d\;\geq \;\alpha \left(e_{+}\right) \end{array}$$

and henceforth designated *e* ↓ *d*.

For a vertex *g* from *G* or *G*′ designate *d*(*g*) a tube that equals *α*(*g*) (if *α*(*g*) is a tube) or the tube incoming in *α*(*g*) (if *α*(*g*) is a vertex). For each vertex from *G*, its clades in *G* and *G*′ are equal. For *g* from *G* the tube *d*(*g*) depends only on clade *g* in *G*; that is, *d*(*g*) is the same in *G* and in *G*′. Note that the triple inequality above is equivalent to

(11) $$\begin{array}{c} d\left(e^{+}\right)>d\geq d\left(e_{+}\right). \end{array}$$

A* paralogous binarization G* ^##^ of *G against S* is a candidate binarization *G*′, in which for each tube *d* the number of entering edges is minimal among all candidate binarizations *G*′. Intuitively, it minimizes the number of paralogs.

A* paralogous binarization G* ^##^ of *G* exists and is produced from the initial *G* with the following iterative procedure. Let a certain *G*′ be already obtained. Choose arbitrarily a polytomous vertex g in *G*′, and let *d*(*g*) produce two child tubes, *d*_1_ and *d*_2_. Divide all children *g*′ of vertex *g* into three parts defined according to the conditions *d*(*g*′) = *d*(*g*), *d*(*g*′) ≤ *d*_1_, *d*(*g*′) ≤ *d*_2_, respectively. The parts are disjoint. If only the first part is nonempty, arbitrarily divide it in two nonempty sets. If the first and at least one of the other two parts are nonempty, the first set coincides with the first part, and the second set is the union of the second and third parts. If the first part is empty, the two sets are the second and third parts, correspondingly; both are nonempty by definition of *d*(*g*). Perform a step of binarization of vertex *g* against partition (*A*, *B*), where *A* is the first set and *B* is the second set. A new *G*′ is thus derived. Apply the procedure until all polytomous vertices are visited; the result, according to Lemma 11, is the paralogous binarization *G* ^##^ of *G* against *S*.

A* bundle of edges* for *d* in *G* is a nonempty maximal on inclusion set of edges *e* in *G* that have the common upper terminus *e* ^+^ (the* vertex parent* of the bundle), and all *e enter d*.

Denote *p*(*G*, *d*) the amount of bundles in *G* for *d*. The* vertex parent* of a bundle *F* is denoted by *F* ^+^. Obviously, a bundle has a unique vertex parent; and vertex parents of different bundles for *d* are different in *G* (and *G* ^##^); edges of different bundles for *d* are incomparable in *G*.

*A complement F*′ of bundle  *F* is a set of edges *e*, for which *e* ^+^ = *F* ^+^ and *e* does not belong to *F*. For the paralogous binarization *G* ^##^, an edge *e* < *F* ^+^ (where *e* and *F* ^+^ are in *G* ^##^) is called a* parent* of bundle *F* in *G* for *d*, if *e*_+_ is the last common ancestor of the lower termini of all edges in *F* and *e*_+_ is not the ancestor of the lower termini of all edges in *F*′.

**Lemma 11**

For any candidate binarization *G*′ and mapping *G*′ into *S*, at least*p*(*G*, *d*) edges enter each tube *d*. For *G* ^##^ (against *S*) and for each bundle (in *G* for* d*) and the mapping *G* ^##^ into *S*, its parent in *G* ^##^ exists and enters the tube *d*. Conversely, each edge entering tube *d* is the parent of a bundle for *d*. Consequently, *G* ^##^ is a paralogous binarization.

**Proof**

The first statement. In the mapping of *G*′ into *S*, each bundle in *G* for *d* induces at least one edge entering *d*; different bundles induce different edges. Indeed, let *e* be any edge in the bundle. Then on a path in *G*′ connecting *e* ^+^ and *e*_+_, there exists an edge in *G*′ that enters *d*. As for any two bundles for *d*, their vertex parents are different; for any two corresponding paths in *G*′, the set of edges in one path does not intersect with the set of edges in another path. Consequently, at least *p*(*G*, *d*) edges enter *d*.

The second statement. Let *F* be a bundle for tube *d*, and *g* = *F* ^+^. By definition of the bundle, *d*(*g*) > *d*, and for all lower termini *g*_*i*_ of edges in *F*, we observe *d*(*g*_*i*_) ≤ *d*. If the vertex *g* is binary or is the superroot, then |*F*| = 1, and the assertion is obvious. Let vertex *g* be polytomous. If |*F*| = 1, the assertion is obvious. Otherwise, consider in *G* ^##^ a maximally long path *L* of vertices *g*_1_ = *g*, *g*_2_,…, *g*_*k*_, where each vertex descends directly from the other and is ancestor of the lower termini of all edges in the bundle *F*. Observe *d*(*g*_*k*_) ≤ *d*; otherwise during partitioning, the set of children of *g*_*k*_ in the constructed *G* ^##^, all children from the bundle *F* belong in one part (the second or the third one), which contradicts the assumption of maximal *L*. It follows that the edge (*g*_*k*−1_, *g*_*k*_) enters the tube *d* and is the parent of the bundle *F*.

The third statement. Let *e* ↓ *d*, where *e* is an edge in *G* ^##^. By constructing the candidate binarization, there exists such vertex *g* in *G* that *g* > *e* > *g*′, where *g*′ is a child of *g* in *G*. Consider the set of children *g*′ of vertex *g*, for which *g*′ < *e* in *G* ^##^. The edges in *G* having lower terminus in this set form a nonempty subset of a certain bundle *F* for *d*, where *F* ^+^ = *g*. Let *e*′ be the bundle parent. Then *e* is comparable with *e*′, and *e*′ enters *d* according to Lemma 11. Any two comparable edges cannot enter the same tube; therefore *e* = *e*′.

The described paralogous binarization procedure runs in linear time.

**Lemma 12**

Let *F*_1_ be a bundle for *d*_1_, let *F*_2_ be a bundle for *d*_2_ (both in *G*), and *d*_1_^+^ = *d*_2_^+^. If *F*_1_^+^ = *F*_2_^+^, then in the paralogous binarization *G* ^##^, the parents of *F*_1_ and *F*_2_ share the common upper terminus. And, conversely, if the parents of *F*_1_ and *F*_2_ share a common upper terminus in *G* ^##^, then *F*_1_^+^ = *F*_2_^+^.

**Proof**

Define with *d* a tube such that *d*_+_ = *d*_1_^+^ = *d*_2_^+^, and with *g* a vertex *F*_1_^+^ = *F*_2_^+^. If *g* is a binary vertex in *G*, the assertion is obvious.

Otherwise, consider in *G* ^##^ a maximally long path *L* of consecutive vertices *g*_1_ = *g*, *g*_2_,…, *g*_*k*_, where each *g*_*i*_ is an ancestor of a set *C* of all lower termini of edges in the union of *F*_1_ and *F*_2_. Observe *d*(*g*_*k*_) = *d*; otherwise during partitioning the set of children of *g*_*k*_ in the constructed *G* ^##^, all its children from *C* would belong in one part (the second or third one), which contradicts the assumption of maximal *L*.

The parents of *F*_1_ and *F*_2_ share a common upper terminus, as in the constructed *G* ^##^ the bundles *F*_1_ and *F*_2_ correspond to the second and third parts of the *g*_*k*_ children set (the first part is empty, as follows from the assumption of maximal *L*). By the procedure, the parts are induced by separate edges, the parents of the corresponding bundles, and the two edges share the common upper terminus.

Prove the converse statement by contradiction. Denote the parents of bundles *F*_1_ and *F*_2_ as *e*_1_ and *e*_2_. Consider in *G* ^##^ a path *p*_1_ connecting *F*_1_^+^ with the lower terminus of an arbitrary edge from *F*_1_ and a path *p*_2_ connecting *F*_2_^+^ with the lower terminus of an arbitrary edge from *F*_2_. Then *p*_1_ contains *e*_1_ and *p*_2_ contains *e*_2_. By our assumption, *F*_1_^+^ ≠ *F*_2_^+^. Consequently, no two edges, one belonging to *p*_1_ and the other belonging to *p*_2_, can share a common upper terminus. The contradiction is obtained.

The number of different *G* ^##^ is exponential of the maximal number of edges *e* in *G* sharing the upper terminus *e* ^+^, for which *d*(*e*_+_) = *d*(*e* ^+^). Importantly, any *G* ^##^ can be legitimately used in Section 2.13, according to Lemma 13. Our algorithm of constructing one *G* ^##^ for any *G* can be easily extended to enumerate any portion of the binarization solutions space.

**Lemma 13**

Embedding costs of all *G* ^##^ against a fixed *S* are equal.

**Proof**

Each paralogous binarization *G* ^##^ possesses the same amount of vertices. Note that in a canonic mapping each edge *e* entering a tube *d* corresponds either to a divergence (if *α*(*e* ^+^) = *d* ^+^) or loss (otherwise). Conversely, a divergence corresponds to a pair of edges with a common upper terminus entering the tubes a with common upper terminus, and a loss corresponds to edge *e* entering tube *d*, where *α*(*e* ^+^) ≠ *d* ^+^. Hence, draw two bijective correspondences:

(1) between divergences and unordered pairs {〈*e*_1_, *d*_1_〉, 〈*e*_2_, *d*_2_〉}, where *i* = 1,2, *e*_*i*_ ↓ *d*_*i*_, *e*_1_^+^ = *e*_2_^+^, and *d*_1_^+^ = *d*_2_^+^;

(2) between losses and pairs 〈*e*, *d*〉, where *e* ↓ *d*, which do not fall in correspondence (1) with any divergence.

According to these correspondences and Lemmas 11-12, in a mapping of a paralogous binarization into *S*, there exist as many divergences as there are unordered pairs of bundles of the form 〈bundle  *F*_1_  for  *d*_1_, bundle  *F*_2_  for  *d*_2_〉 in *G*, where *d*_1_^+^ = *d*_2_^+^, *F*_1_^+^ = *F*_2_^+^. Other nonleaf vertices are duplications; therefore their amount does not depend on *G* ^##^. The amount of losses is also *G* ^##^-independent; according to the correspondences above and Lemma 11, there exist as many losses as there are bundles that do not fall in the correspondence with any divergence.

**Lemma 14**

In a paralogous binarization *G* ^##^, let an edge *e* be a parent of a bindle *F*. The number of leaves contained in the clade of *e*_+_ in *G* ^##^ does not depend on *G* ^##^.

**Proof**

By definition of the bundle parent, the set of leaves contained in *G* ^##^ below *e* is the set of leaves contained in *G* below the edges of bundle *F*. This set depends only on *G* and *F*.

For canonic mappings of *G* ^##^ (against *S*) into *S*, hold the following analogs of Lemmas 5–7.

**Lemma 15**

In a canonic mapping of *G* ^##^ into *S*, each leaf tube *d* of species *s* contains the amount of duplications equal to the difference between the total amount *L* of leaves below the edges of the bundles from *G* for *d* and the amount of all bundles for *d* in *G*.

**Proof**

Denote this amount of duplications Par′(*G*, *s*). In a canonic mapping of *G* ^##^ into *S*, the edges parental to the bundles for a leaf tube *d* enter the tube *d*, and all nonleaf vertices in the tree *G* ^##^ lower to these edges are duplications. By Lemma 14, for each such bundle *F*, there are *L* leaf vertices lower to the parent of *F*. A binary tree contains *n* − 1 internal vertices compared with the number *n* of leaves; therefore, the number of duplications is also *n* − 1 of the number *n* of edges in a bundle.

**Lemma 16**

Fix a polytomous tree *G* over a subset of *V*_0_. Let species trees *S*_1_ and *S*_2_ be both defined over *V*_0_, each containing a certain subtree *S*. The total costs of all events in the mappings of *G* ^##^ into *S*_1_ and*G* ^##^ into *S*_2_, having place in *S*, are equal. In other words, the total cost depends only on the subtree *S* and not on its complement.

**Proof**

In a canonic mapping of *G* ^##^ into *S*_1_ or *S*_2_, the edges of tree *G* ^##^, the parents of the bundles for the root tube *d* in a subtree *S*, enter the tube *d*. All vertices below these edges map into *S*. Conversely, if a vertex *g* maps into *S*, then on the path connecting it with the superroot, there exists an edge entering *d* and, by Lemma 11, being a parent of a bundle for *d*. If an edge *e* from *G* ^##^ is parental to a bundle for *d*, then by Lemma 14 the set of leaves below *e* is defined by the bundle and does not depend on *G* ^##^. The number of vertices in a binary subtree is determined by the number of leaves. Consequently, the amount of vertices mapped into *S* does not depend on *G* ^##^. According to correspondence (1) stated in the proof of Lemma 1 and to Lemma 11, the amount of divergences in these vertices is exactly the amount of the unordered pairs 〈bundle  *F*_1_  for  *d*_1_, bundle  *F*_2_  for  *d*_2_〉 in *G*, where *F*_1_^+^ = *F*_2_^+^, *d*_1_^+^ = *d*_2_^+^, and *d*_1_^+^ lies in *S*. Other nonleaf vertices are duplications. According to correspondence (2) stated in the proof of Lemma 1 and to Lemma 11, the number of losses in *S* is also *G* ^##^-independent and is exactly the number of bundles for tubes *d*, which do not fall in a correspondence with any divergence where *d* ^+^ lies in *S*.

**Lemma 17**

Fix a polytomous tree *G* over a subset of *V*_0_. Let *V* be a subset of  *V*_0_, and a species tree *S*_1_ over *V*_0_ contains a subtree *T*_1_ over *V*. Let a tree *S*_2_ be derived from *S*_1_ by substituting the subtree *T*_1_ with a subtree *T*_2_ over *V*. The total costs of all events in the mappings of *G* ^##^ into *S*_1_ and *S*_2_ having place in the complements of *T*_1_ in *S*_1_ and *T*_2_ in *S*_2_ are equal. In other words, the total event cost does not depend on a subtree.

**Proof**

The mapping *α* maps into each *T*_*i*_ vertices in a tree *G* ^##^ that are below the edges parental to the bundles for the root tube *d* in *T*_*i*_. By definition of the bundle, the set of such bundles depends only on the clade of *d*_+_ that does not depend on index *i*. According to the argument in the proof of Lemma 16, the same amount of such vertices is mapped in each *T*_*i*_, regardless of *G* ^##^. Consequently, the complement of *T*_*i*_ receives the same amount of vertices. Among these vertices, the number of divergences equals exactly the number of the unordered pairs 〈bundle  *F*_1_  for  *d*_1_, bundle  *F*_2_  for  *d*_2_〉 in *G*, where *F*_1_^+^ = *F*_2_^+^, *d*_1_^+^ = *d*_2_^+^, and *d*_1_^+^ does not lie in *S*_*i*_. Other nonleaf vertices are duplications. The number of losses in *S* is also *G* ^##^-independent and is exactly the number of bundles for tubes *d*, which do not fall in a correspondence with any divergence where *d* ^+^ does not lie in *T*_*i*_.

### 2.13. The Second Problem for a Fixed Set of Polytomous Gene Trees

The general problem for a given set of polytomous gene trees *G*_*j*_ is to find a species tree *S* ^#^ that minimizes the total sum (over binarizations *G*_*j*_^#^of all *G*_*j*_) of the mappings of *G*_*j*_^#^ into *S* ^#^. The unconditional (absolute) problem imposes no constraint on the solution space. In the conditional problem, the search space of trees (including *S* ^#^) is limited to the clades belonging to a prefixed parameter *P*; all clades from all *G*_*j*_ are included in *P* by default. The found binarization *G*_*j*_^#^ may not be unique, but its choice does not affect the functional. The authors are only aware of an exponential complexity algorithm that solves both the unconditional and conditional problems. However, such complexity renders it of little use.

We formulate a simplification of the conditional problem; paralogous binarizations *G*_*j*_^##^ are used instead of arbitrary candidate binarizations as described in Section 2.12.

A simplified problem is to construct a tree *S* ^##^ (also containing clades from the set *P*) that minimizes the functional *c*({*G*_*j*_^##^(*S*)}, *S*, {*f*_*j*_}), where *G*_*j*_^##^(*S*) is any paralogous binarizations of the initial trees *G*_*j*_ against *S*. By Lemma 13, the functional value is independent of the choice of *G*_*j*_^##^(*S*).

This *S* ^##^ does not generally provide a global solution but can be useful, as paralogous binarizations are often biologically realistic.

Our solving algorithm for the simplified problem is similar to the case of binary trees and consists of two phases, with Phase 2 being identical. Phase 1 uses the same induction to build basic trees *S*(*V*). The start of induction is identical to the binary case, with replacing ∑_*j*_Par(*G*_*j*_, *s*) to ∑_*j*_Par′(*G*_*j*_, *s*). In the induction step, the only difference with the binary case is the calculation of the cost of the events from the third group. By enumerating all vertices in all given *G*_*j*_, compute the numbers of all bundles in {*G*_*j*_} for each *d*, *d*_1_, and *d*_2_ and denote those numbers *n*, *n*_1_, and *n*_2_, respectively. Analogously find the number *k* of pairs of all bundles of the form 〈bundle  *F*_1_  for  *d*_1_, bundle  *F*_2_  for  *d*_2_〉 in {*G*_*j*_} for each *d*_1_ and *d*_2_, where *F*_1_^+^ = *F*_2_^+^. Find values *n*_*i*_ and *k* in {*G*_*j*_}, for which (i) the root clade intersects with both sets *V*_1_ and *V*_2_, and (ii) the root clade intersects with one of the sets and not with the other. Designate *n*_*i*_′, *k*′ the numbers for (i), and *n*_*i*_′′,*k*′′ the numbers for (ii).

Define

(12) c(V,V1,V2)=c(V1)+c(V2)+cdiv⁡·k+cdup·(n1+n2−n−k)+clos1·(n1′+n2′−2k′)+clos2·(n1′′+n2′′−2k′′).

*Justification of the Algorithm*. Let *S* be an arbitrary species tree over *V*_0_ that includes a subtree *S*(*V*). The total cost of events in *S*(*V*) undercanonic mappings of all *G*_*j*_^##^ (against *S*) into *S* is* designated c*(*V*), analogously to the binary case. Obviously, if *V* = *V*_0_, then *c*(*V*_0_) = *c*({*G*_*j*_^##^}, *S*, {*f*_*j*_}). According to Lemma 16, *c*(*V*) also depends on the subtree *S*(*V*) only and not on its complement (in the tree *S*).  Theorem 18 is analogous to Theorem 8.

**Theorem 18**

A basic tree *S*(*V*) globally minimizes the functional *c*(*V*) in the conditional problem for *V*, if a solution exists.

**Proof**

For a singleton set *V*, the assertion of the theorem follows from Lemma 15.

According to Lemma 11, the number of edges entering a tube in a canonic mapping of *G* ^##^ into *S* equals the number of the bundles in *G* for this tube. The mapping in *d* or *r* involves exactly the vertices of *G* ^##^ that are both the descendants of one of the edges entering *d* and ancestors of at least one edge entering *d*_1_ or *d*_2_. Obviously, there are *n*_1_ + *n*_2_ − *n* such vertices. Among them, the number of divergences (mappings in *r*) is exactly the number of pairs of the bundles 〈bundle  *F*_1_  for  *d*_1_, bundle  *F*_2_  for  *d*_2_〉 in *G*, where *F*_1_^+^ = *F*_2_^+^. The number of losses in *r* is exactly the number of bundles for *d*_1_ or *d*_2_ that do not fall in a correspondence with any divergence. Therefore, under *k* divergences, there exist *n*_1_ + *n*_2_ − *n* − *k* duplications and *n*_1_ + *n*_2_ − 2*k* losses. Consequently, the value *c*_div⁡_ · *k* + *c*_dup_ · (*n*_1_ + *n*_2_ − *n* − *k*) + *c*_los1_ · (*n*_1_′ + *n*_2_′ − 2*k*′) + *c*_los2_ · (*n*_1_′′ + *n*_2_′′ − 2*k*′′) is the total cost of events in the third group.

Further justification of the algorithm is identical to the binary case (considering Lemma 17). The remark to the proof of Theorem 8 and modification of Phase 1 (refer to Section 2.11) are still valid.

The solution of the simplified conditional problem is obtained. The running complexity of the algorithm has the same order as specified in Theorem 8.
